# One‐Pot Hetero‐Di‐*C*‐Glycosylation of the Natural Polyphenol Phloretin by a Single *C*‐Glycosyltransferase With Broad Sugar Substrate Specificity

**DOI:** 10.1002/bit.28948

**Published:** 2025-02-07

**Authors:** Tuo Li, Annika J. E. Borg, Leo Krammer, Rolf Breinbauer, Bernd Nidetzky

**Affiliations:** ^1^ Institute of Biotechnology and Biochemical Engineering Graz University of Technology Graz Austria; ^2^ Austrian Centre of Industrial Biotechnology (acib) Graz Austria; ^3^ Institute of Organic Chemistry Graz University of Technology Graz Austria

**Keywords:** *C*‐glycosyltransferase, cascade reaction, hetero‐di‐*C*‐glycosylation, natural polyphenol, one‐pot reaction, sugar substrate specificity

## Abstract

The structural motif of hetero‐di‐*C*‐glycosyl compound is prominent in plant polyphenol natural products and involves two different glycosyl residues (e.g., β‐d‐glucosyl, β‐d‐xylosyl) attached to carbons of the same phenolic ring. Polyphenol hetero‐di‐*C*‐glycosides attract attention as specialized ingredients of herbal medicines and their tailored synthesis by enzymatic *C*‐glycosylation is promising to overcome limitations of low natural availability and to expand molecular diversity to new‐to‐nature glycoside structures. However, installing these di‐*C*‐glycoside structures with synthetic precision and efficiency is challenging. Here we have characterized the syntheses of *C*‐β‐galactosyl‐*C*‐β‐glucosyl and *C*‐β‐glucosyl‐*C*‐β‐xylosyl structures on the phloroglucinol ring of the natural polyphenol phloretin, using kumquat (*Fortunella crassifolia*) *C‐*glycosyltransferase (*Fc*CGT). The *Fc*CGT uses uridine 5'‐diphosphate (UDP)‐galactose (5 mU/mg) and UDP‐xylose (0.3 U/mg) at lower activity than UDP‐glucose (3 U/mg). The 3'‐*C*‐β‐glucoside (nothofagin) is ~10‐fold less reactive than non‐glycosylated phloretin with all UDP‐sugars, suggesting the practical order of hetero‐di‐*C*‐glycosylation as *C*‐galactosylation or *C*‐xylosylation of phloretin followed by *C*‐glucosylation of the resulting mono‐*C*‐glycoside. Each *C*‐glycosylation performed in the presence of twofold excess of UDP‐sugar proceeds to completion and appears to be effectively irreversible, as evidenced by the absence of glycosyl residue exchange at extended reaction times. Synthesis of *C*‐β‐glucosyl‐*C*‐β‐xylosyl phloretin is shown at 10 mM concentration in quantitative conversion using cascade reaction of *Fc*CGT and UDP‐xylose synthase, allowing for in situ formation of UDP‐xylose from the more expedient donor substrate UDP‐glucuronic acid. The desired di‐*C*‐glycoside with Xyl or Gal was obtained as a single product of the synthesis and its structure was confirmed by NMR.

## Graphical abstract

Here, an efficient system for synthesizing high‐value polyphenol hetero‐di‐*C*‐glycosides with two distinct *C*‐glycosyl residues was established using a two‐step glycosylation catalyzed by *C*‐glycosyltransferase in one pot. The desired *C*‐β‐galactosyl‐*C*‐β‐glucosyl and *C*‐β‐glucosyl‐*C*‐β‐xylosyl phloretin were obtained at concentrations of 0.6 and 6 g/L, respectively, with quantitative conversion. The in‐situ formation of UDP‐xylose from UDP‐glucuronic acid via UDP‐xylose synthase, along with the regeneration of UDP‐glucose through sucrose synthase, was carried out during the cascade synthesis of *C*‐β‐glucosyl‐*C*‐β‐xylosyl phloretin.

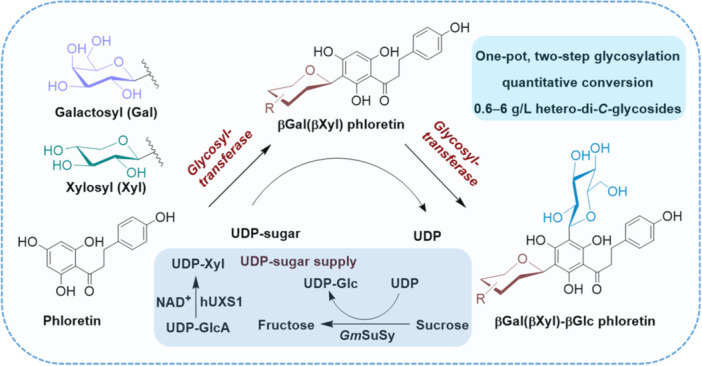

**Abbreviations**
CGTs: C‐glycosyltransferases
*Fc*CGT: *C*‐glycosyltransferase from kumquat (*Fortunella crassifolia*)
hUXS1: UDP‐xylose synthase from *Homo sapiens*
*Gm*SuSy: sucrose synthase from soybean (*Glycine max*)
HPCD: 2‐hydroxypropyl‐β‐cyclodextrin
UDP‐Gal: UDP‐galactose
UDP‐Xyl: UDP‐xylose
UDP‐Glc: UDP‐glucose
UDP‐GlcA: UDP‐glucuronic acid
UDP: uridine‐5'‐diphosphate
βGal‐βGlc phloretin: 3'‐*C*‐β‐galactosyl‐5'‐*C*‐β‐glucosyl phloretin
βGlc‐βXyl phloretin: 3'‐*C*‐β‐glucosyl‐5'‐*C*‐β‐xylosyl phloretin

Aryl di‐*C*‐glycosylation is a specialized type of structural modification in natural products, plant polyphenols in particular (Liu 2022). The known di‐*C*‐glycosides of polyphenols feature two *C*‐glycosyl residues attached at different positions on the same phenolic ring (Kitamura et al. 2018). Homo‐di‐*C*‐glycosides comprise two of the same glycosyl residues, usually β‐glucosyl (Liu 2022), as for example in the 3',5'‐di‐*C*‐β‐glucosyl derivative of the important plant chalcone phloretin (**1a**, Figure 1a). Hetero‐di‐*C*‐glycosides involve two different *C*‐glycosyl residues, typically β‐glucosyl in combination with another sugar such as d‐xylosyl, l‐arabinosyl or l‐rhamnosyl (Shie et al. 2010). For example (Figure 1a), the common flavone apigenin is naturally found hetero‐6,8‐di‐*C*‐glycosylated with βGlc‐βXyl (vicenin‐3, **2a**) (Jeong et al. 2017), βGlc‐αAra (schaftoside, **3a**) (Jeong et al. 2017) or βGlc‐αRha (violanthin, **4a**) (Han et al. 2023) structures. The polyphenol di‐*C*‐glycosides are the most powerful antioxidants and exhibit unique biological activities (e.g., anti‐inflammatory) considered important in herbal medicine and health‐related nutrition (Aidhen et al. 2022). Isoschaftoside (6‐αAra‐8‐βGlc‐apigenin, **3b**; Supporting Information S1: Figure 1) is an allelochemical considered for crop protection (Hooper et al. 2010). Compound **1a** is suggested as a quality‐control marker for the shelf‐life of herbal tea‐based beverages (Human et al. 2021). Exploring new applications of polyphenol di‐*C*‐glycosides is often hindered by low availability from their natural plant sources (Shie et al. 2010). Chemical synthesis can be useful to overcome the limitation. Despite notable progress made at the front of the homo‐di‐*C*‐glycosyl compounds (Ho et al. 2016), installing hetero‐di‐*C*‐glycosyl motifs with synthetic precision and efficiency remains a challenge for existing methods of synthesis (Sato and Koide 2010).

**Figure 1:**
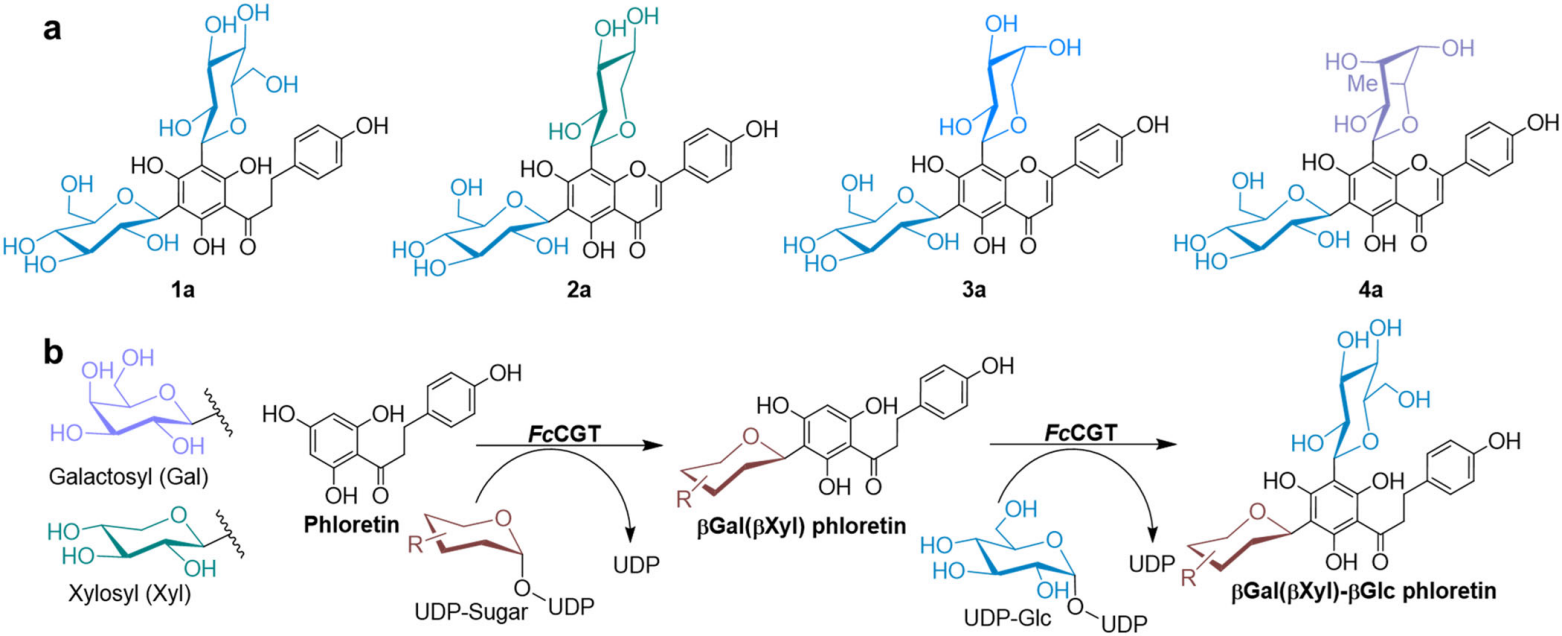
(a) Structure of compound **1a** (3',5'‐di‐*C*‐β‐glucosyl phloretin), **2a** (vicenin‐3), **3a** (schaftoside) and **4a** (violanthin). (b) Reaction scheme for the two‐step synthesis of βGal‐βGlc and βGlc‐βXyl phloretin by a di‐CGT (*Fc*CGT).

Direct *C*‐glycosylation of the unprotected polyphenol from a sugar nucleotide donor is a promising strategy of applied bio‐catalysis for *C*‐glycoside synthesis (Chong et al. 2022; Putkaradze et al. 2021). The enzymes used are specialized glycosyltransferases (EC 2.4) that target an aryl carbon of the polyphenol aglycone (Tegl and Nidetzky 2020). A rich diversity of natural and engineered *C*‐glycosyltransferases (CGTs) has recently become available (Dai et al. 2021; Li et al. 2024; Putkaradze et al. 2023). By combining CGTs of complementary sugar nucleotide specificity for hetero‐di‐*C*‐glycosylation, the di‐*C*‐glycosyl compounds **2a** (Z. Chen et al. 2021; Feng et al. 2021), **3a** (Z. Chen et al. 2021; Feng et al. 2021; Wang et al. 2020) and **4a** (Han et al. 2023) as well as their corresponding isomers (Supporting Information S1: Figure 1; vicenin‐1, **2b**; isoschaftoside **3b**; isoviolanthin, **4b**) were prepared. CGTs performing an iterative di‐*C*‐glycosylation of polyphenol substrates (di‐CGTs) were shown for homo‐di‐*C*‐glucoside synthesis (Li et al. 2023). The broad specificity of some di‐CGTs for their sugar nucleotide substrates enabled the instalment of hetero‐di‐*C*‐glycosyl motifs (e.g., βGlc‐βXyl) on different polyphenol aglycones, including phloretin (D. Chen et al. 2018; Zhang et al. 2020). However, while the use of a single “promiscuous” di‐CGT is attractive for the synthesis in principle, it also involves challenges of engineering and control of the two‐step *C*‐glycosylation, to give a defined single hetero‐di‐*C*‐glycosyl product in useful yield and productivity. Kinetic and thermodynamic analysis of the component steps of the overall hetero‐di‐*C*‐glycosylation is critical for enhanced process understanding as the basis for optimization and scale‐up. Here, we show the di‐CGT from *Fortunella crassifolia* (kumquat; *Fc*CGT) (Ito et al. 2017) for hetero‐di‐*C*‐glycosylation of phloretin (Figure 1b) and present a detailed characterization of biocatalytic reactions in one pot, to deliver the βGal‐βGlc and βGlc‐βXyl di‐*C*‐glycosyl derivatives with high purity and in high overall transformation efficiency. Among other choices of di‐CGT (e.g., D. Chen et al. 2018; Zhang et al. 2020), the *Fc*CGT was used here due to the proven performance of the enzyme in the synthesis of the 3',5'‐di‐*C*‐β‐glucoside of phloretin (Li et al. 2023).

To select a suitable sequence of steps for hetero‐di‐*C*‐glycosylation (Supporting Information S1: Figure 2), we determined the specific activities of *Fc*CGT (Supporting Information S1: Table 1) for reaction with the different sugar nucleotides (each 2.0 mM) using phloretin or nothofagin (3'‐*C*‐β‐glucosyl phloretin) as the acceptor (1.0 mM). The activity followed the order UDP‐glucose (3.02 U/mg; Li et al. 2023), UDP‐xylose (325 ± 21 mU/mg) and UDP‐galactose (4.60 ± 0.42 mU/mg) when phloretin was used. Kinetic parameters for the three sugar nucleotides (Supporting Information S1: Table 2) confirmed the donor substrate specificity of *Fc*CGT for *C*‐glycosylation of phloretin. In terms of *k*_cat_/*K*_M_, UDP‐glucose was preferred 21‐fold over UDP‐xylose and 3.8 × 10^3^‐fold over UDP‐galactose. The variation in the *k*_cat_/*K*_M_ was mainly due to a change of comparable magnitude in the *k*_cat_. The *K*_M_ increased in the order UDP‐glucose, UDP‐xylose (2.2‐fold) and UDP‐galactose (7.8‐fold). Changes in the *K*_M_ were however small compared to changes in *k*_cat_. With nothofagin as the acceptor, the activity of *Fc*CGT was generally about one magnitude order lower, that is, UDP‐glucose (0.41 U/mg; Li et al. 2023), UDP‐xylose (26.3 ± 0.99 mU/mg) and UDP‐galactose (0.57 ± 0.05 mU/mg). From reaction time course studies shown later, we further determined that the activity for *C*‐glycosylation from UDP‐glucose of the 3'‐*C*‐β‐xylosyl and 3'‐*C*‐β‐galactosyl derivatives of phloretin was 167 (± 1) and 5.05 ( ± 0.49) mU/mg, respectively. The results reveal the clear preference of *Fc*CGT for reaction with UDP‐glucose compared to UDP‐xylose and UDP‐galactose, suggesting that the *C*‐β‐glucosylation be performed only as the second reaction step, once the attachment of the *C*‐β‐xylosyl or *C*‐β‐galactosyl residue was completed. The alternative order of hetero‐di‐*C*‐glycosylation with *C*‐β‐glucosylation of phloretin as the first step was not considered promising for reasons of low *Fc*CGT activity and possible competition for the usage of sugar nucleotide donor when in a one‐pot process excess UDP‐glucose was carried over from the first into the second glycosylation step. Interestingly, whereas the 3'‐*C*‐β‐xylosyl substitution on phloretin was tolerated well by *Fc*CGT in the subsequent *C*‐β‐glucosylation from UDP‐glucose, the 3'‐*C*‐galactosyl substitution caused massive (~80‐fold) loss of activity compared to the reference reaction with nothofagin as acceptor. Considering the rather permissive specificity of *Fc*CGT for the acceptor substrate used, the pronounced discrimination between 3'‐*C*‐β‐glucosyl and 3'‐*C*‐β‐galactosyl phloretin as an acceptor for *C*‐glucosylation from UDP‐glucose was surprising.

Figure 2 summarizes the results of a comprehensive time course analysis of the sequential *C*‐β‐galactosylation and *C*‐β‐glucosylation of phloretin. The components of the reaction (phloretin and *C*‐glycosyl compounds thereof, Figure 2a; UDP and UDP‐sugars, Figure 2c) were well‐separated and quantified by HPLC. The reaction proceeding in two steps—phloretin → 3'‐*C*‐β‐galactosyl phloretin → 3'‐*C*‐β‐galactosyl‐5'‐*C*‐β‐glucosyl phloretin—is shown in Figure 2b. The di‐*C*‐glycosylated phloretin was isolated and its structure was confirmed by LC‐MS and NMR (Supporting Information S1: Figure 3–6). The *C*‐β‐galactosylation of phloretin required ∼18 h to complete. Interestingly, despite the similar initial rates of both glycosylation steps (∼1.4 mM/h), the *C*‐β‐glucosylation of 3'‐*C*‐β‐galactosyl phloretin was completed in just one‐ninth of the time. The kinetic characteristics of *Fc*CGT appear to be more favorable for *C*‐β‐glucosylation than *C*‐β‐galactosylation. The substrate affinity (1/*K*_M_) was ~8‐fold higher for UDP‐glucose than UDP‐galactose (Supporting Information S1: Table 2), which might explain the pronounced slowing of the *C*‐β‐galactosylation as the conversion progresses. The consumption of UDP‐galactose and UDP‐glucose was matched (within a limit of ≤ 10%) to the formation of the mono‐ and di‐*C*‐glycosylated phloretin, respectively (Figure 2d). UMP is the main product of chemical degradation of UDP‐sugars. It was released in low amount (≤ 5% of sugar nucleotide used). Overall, these results suggest a highly chemo‐selective transformation catalyzed by *Fc*CGT in which alternative uses of the sugar nucleotides in undesired side reactions, enzymatic hydrolysis in particular, are prevented effectively. With various glycosyltransferases, the donor substrate hydrolysis can become pronounced when the enzymes are required to work with slow substrates (McArthur and Chen 2016).

**Figure 2:**
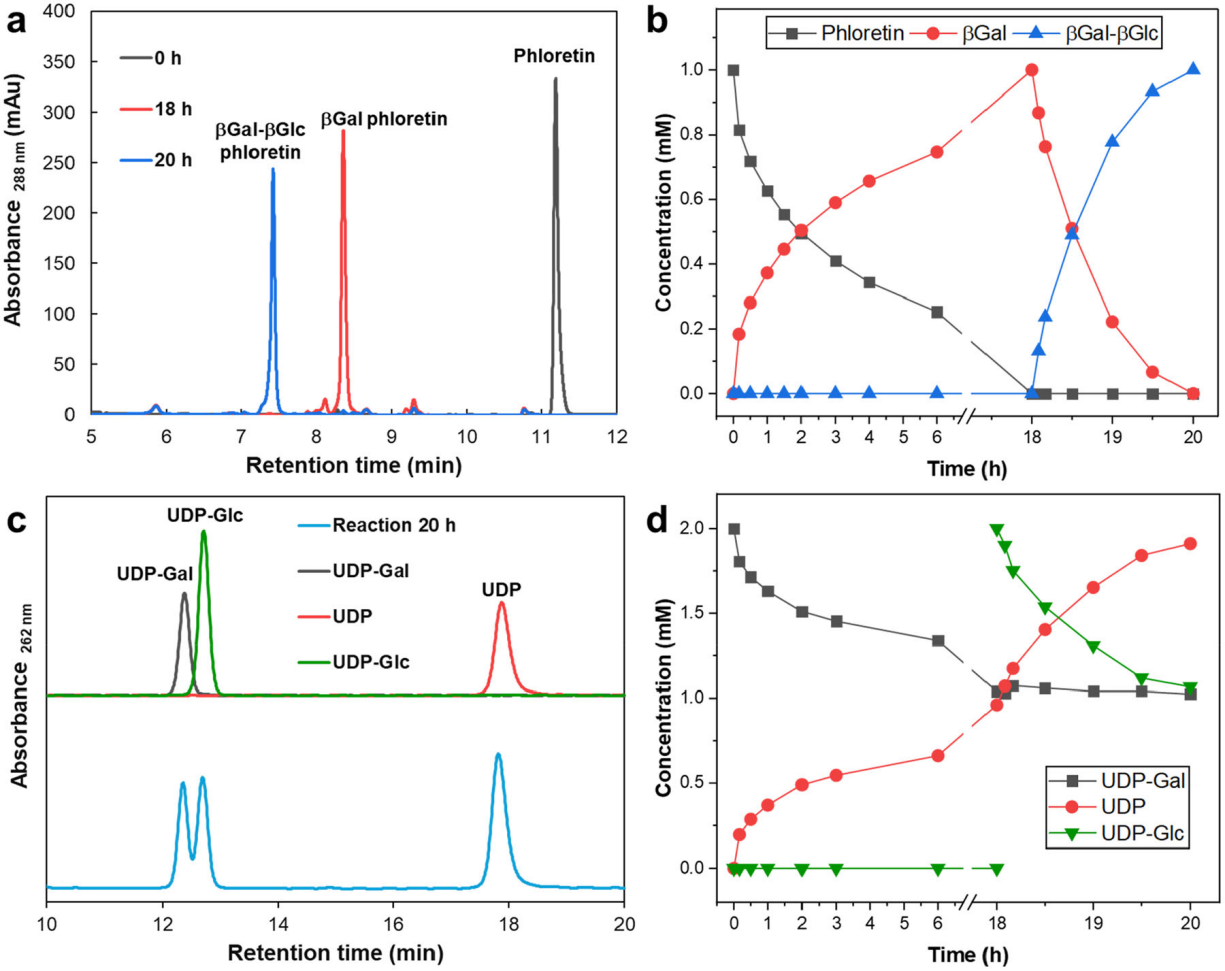
HPLC chromatograms and time courses for acceptor substrate/products (a and b) and donor/products (c and d) in the synthesis of βGal‐βGlc phloretin through two‐step *Fc*CGT reactions. Reactions (100 μL) contained 1.0 mM phloretin, 2.0 mM UDP‐Gal, 5.0 mg/mL *Fc*CGT, 10 mM 2‐mercaptoethanol, in potassium phosphate buffer (50 mM, pH 8.0), and were carried out at 30°C without agitation. After 18 h, 2.0 mM UDP‐Glc (final concentration) was added.

Both *C*‐glycosylations of phloretin were quantitative within limits of error, indicating a strong thermodynamic driving force of substrate conversion. Despite the fact that UDP accumulated in the reaction mixture and both UDP‐sugars were present in twofold excess over phloretin, no glycosyl exchange reactions (e.g., *C*‐β‐galactosyl → *C*‐β‐glucosyl) occurred throughout the whole process. Considering the much higher activity of *Fc*CGT for *C*‐β‐glucosylation of the 3'‐*C*‐β‐glucosylated compared to the *C*‐β‐galactosylated phloretin, one would expect the gradual conversion of the initial 3'‐*C*‐β‐galactosyl‐5'‐*C*‐β‐glucosyl phloretin product into 3',5'‐di‐*C*‐β‐glucosyl phloretin in the case that the *C*‐β‐galactosylation was only slightly reversible under the conditions used. However, a single well‐defined hetero‐di‐*C*‐glycosylated product (βGal‐βGlc) was obtained, suggesting that both *C*‐glycosylations of the phloretin were effectively irreversible. Additional time course studies corroborated the conclusions from activity measurements that the reaction sequence (Supporting Information S1: Figure 7), *C*‐β‐galactosylation after *C*‐β‐glucosylation, was unsuitable for practical synthesis of the hetero‐di‐*C*‐glycosyl product. Not only was the *C*‐β‐galactosylation of nothofagin extremely slow (∼ 23% conversion after 24 h), but also the use of UDP‐glucose in the first *C*‐glucosylation had to be strictly limited to avoid byproduct formation from homo‐di‐*C*‐glucosylation of the phloretin.

Synthesis of the βGlc‐βXyl di‐*C*‐glycosyl phloretin is shown in Figure 3. The precise analytical tracking of the conversion of phloretin (Figure 3a) and sugar nucleotides (Figure 3c) enabled a comprehensive characterization of the reaction over time. The *C*‐β‐xylosylation was completed in ∼2 h (Figure 3b). The subsequent *C*‐β‐glucosylation proceeded at a similar rate and gave the hetero‐di‐*C*‐glycosylated phloretin in quantitative conversion. The product was isolated and its structure was confirmed by LC‐MS and NMR (Supporting Information S1: Figures 8–11). Despite the use of a twofold molar excess of UDP‐xylose in the first *C*‐glycosylation, the reaction did not proceed to the di‐*C*‐xylosyl compound. The promiscuous *F*cCGT evidently differs from specialized *C*‐pentosyltransferases (e.g., *Os*UGT708A40 from *Oryza sativa*) catalyzing the *C*‐β‐xylosylation of aryl mono‐*C*‐xylosides (Z. Chen et al. 2021). Figure 3d shows that the UDP‐xylose consumption slowed down substantially when the phloretin conversion into the mono‐*C*‐xylosyl compound reached ∼75%. Addition of UDP‐glucose (2.0 mM) resulted in the complete arrest of utilization of UDP‐xylose (Figure 3d). Only a single product, 3'‐*C*‐β‐glucosyl‐5'‐*C*‐β‐xylosyl phloretin, was obtained. Note: the phloroglucinol ring is symmetrical. The numbering of the aryl carbon to which the *C*‐β‐glucosyl residue is attached in the di‐*C*‐glycosyl compound (3' or 5') is a matter of convention of the chemical nomenclature. As in the synthesis of the βGal‐βGlc phloretin, the utilization of UDP‐xylose and UDP‐glucose was matched exactly to the release of the mono‐ and di‐*C*‐glycosylated phloretin, respectively (Figures 3b and 3d). The absence of *C*‐glycosyl residue exchange (e.g., βXyl by βGlc) indicated that both *C*‐glycosylations were effectively irreversible. As already anticipated from the low activity of *Fc*CGT for glycosyl transfer from UDP‐xylose to nothofagin, synthesis of βGlc‐βXyl phloretin via intermediary nothofagin was rather inefficient, rendering it not practically useful (Supporting Information S1: Figure 12).

**Figure 3:**
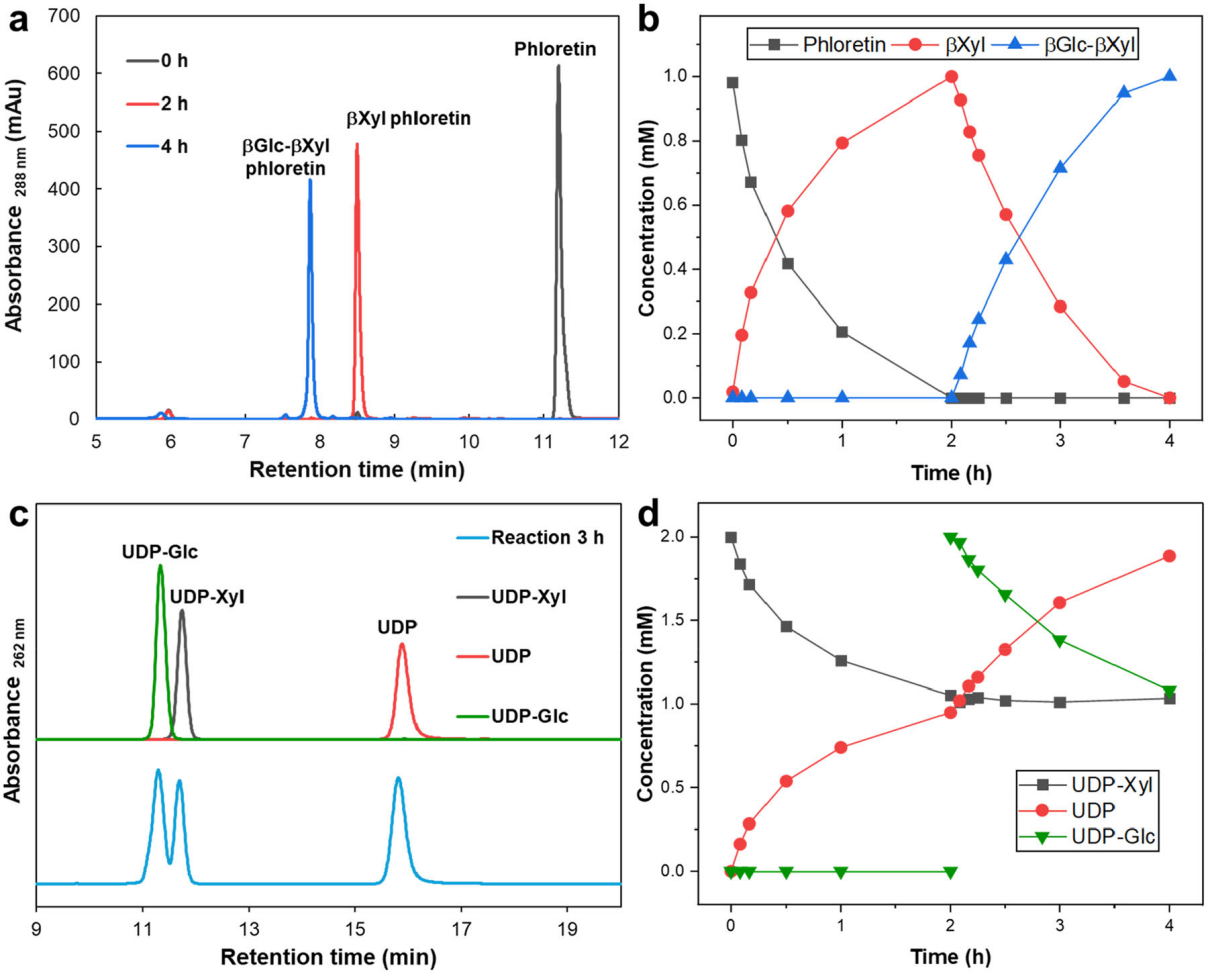
HPLC chromatograms and time courses in the two‐step synthesis of βGlc‐βXyl phloretin through single *Fc*CGT reactions. Overlay of HPLC chromatograms (a) and time course (b) for phloretin, βXyl phloretin and βGlc‐βXyl phloretin. Overlay of HPLC chromatograms (c) and time course (d) for UDP‐Xyl, UDP and UDP‐Glc. Reactions (100 μL) contained 1.0 mM phloretin, 2.0 mM UDP‐Xyl, 0.10 mg/mL *Fc*CGT, 10 mM 2‐mercaptoethanol. After 2 h, the addition of 2.0 mM fresh UDP‐Glc was performed.

Considering synthesis of βGlc‐βXyl phloretin at 10‐fold enhanced concentration, we realized restriction from UDP‐xylose used directly as substrate. To advance the UDP‐xylose supply, we targeted its formation in situ from UDP‐glucuronic acid, using decarboxylation by UDP‐xylose synthase (hUXS1; Figure 4a) (Eixelsberger et al. 2012). The literature reports different routes to UDP‐xylose (for review, see: Frohnmeyer and Elling 2023; Eixelsberger and Nidetzky 2014; Wang et al. 2018; Shi et al. 2022; Wang et al. 2022). The hUXS1 reaction was preferred at this stage because it requires only a single enzyme and can be accommodated readily with the *Fc*CGT reaction into a one‐pot cascade transformation. UDP‐glucuronic acid was hardly used by *Fc*CGT for phloretin glycosylation (≤ 2% conversion in 6 h; Supporting Information S1: Figure 13). Figure 4b shows the reaction of phloretin (1.0 mM). The phloretin conversion into the 3'‐*C*‐β‐xylosyl compound was complete, the productivity was about half that obtained in the reaction with UDP‐xylose used directly (Supporting Information S1: Table 3). The use of UDP‐sugars was again precisely matched to the release of mono‐ and di‐*C*‐glycosylated phloretin (Figure 4c). At the endpoint of *C*‐β‐xylosylation of phloretin (5 h), the conversion of UDP‐glucuronic acid stopped and a constant ratio of UDP‐xylose and UDP‐glucuronic acid (∼1.58 = 0.68 mM/0.43 mM) remained over time. The decarboxylation of UDP‐glucuronic acid is an irreversible process (Eixelsberger et al. 2012). Arrest of the hUXS1 reaction at the partial conversion of UDP‐glucuronic acid must therefore have a kinetic origin. The human hUXS1 here used was active for 24 h at least (Eixelsberger and Nidetzky 2014), excluding enzyme inactivation as the decisive factor. However, UDP‐xylose causes strong inhibition of the enzyme through complex mechanisms of binding.

**Figure 4:**
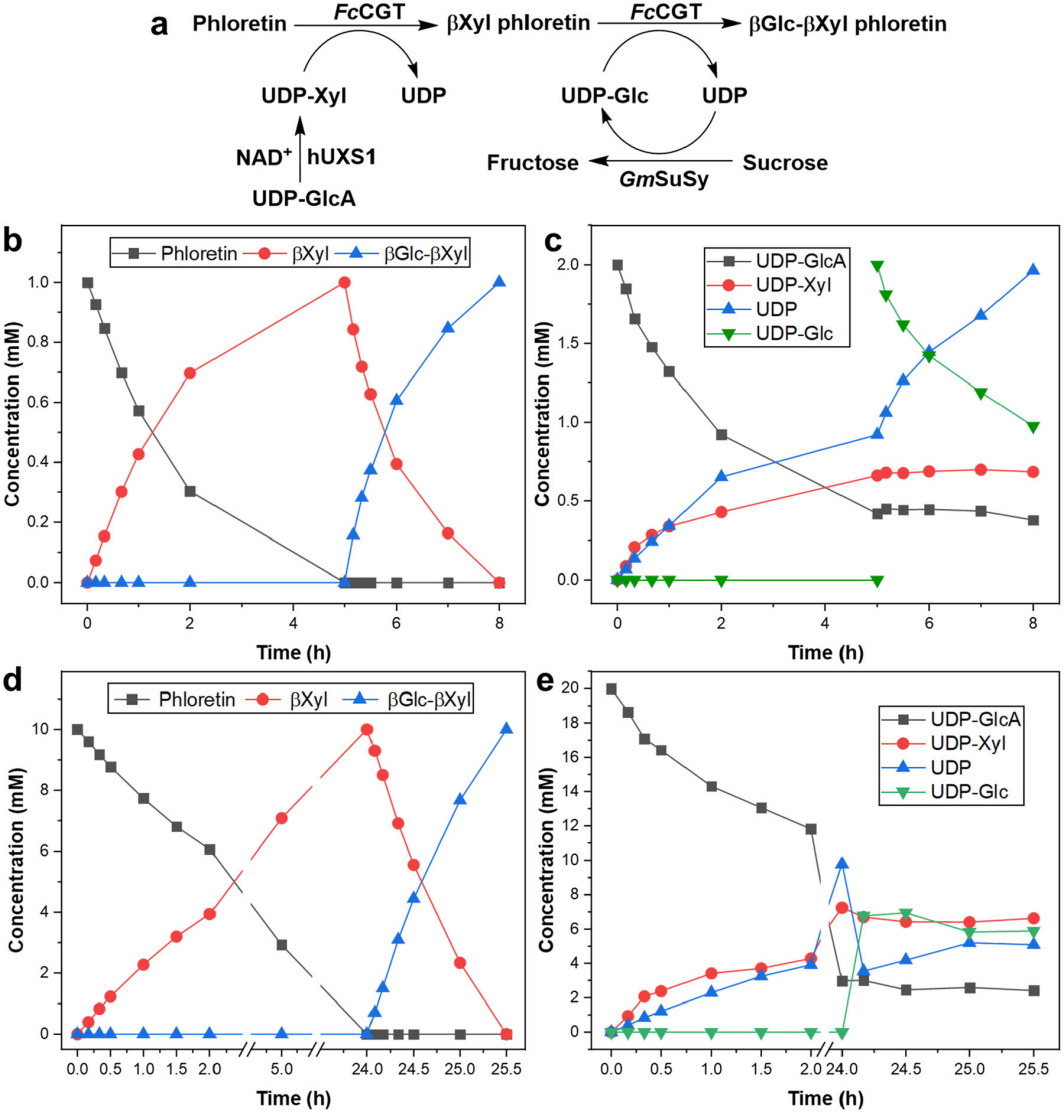
Scheme of the cascade reaction used (a) and time courses b–e) for the two‐step synthesis of βGlc‐βXyl phloretin through *Fc*CGT/hUXS1 one‐pot transformation. Time courses for phloretin/products (b) and UDP‐sugars/UDP (c) in 1.0 mM phloretin reactions. Time courses for phloretin/products (d) and UDP‐sugars/UDP (e) in 10 mM phloretin reactions. The reaction conditions were described in the MATERIALS under “Cascade reactions for the synthesis of βGlc‐βXyl phloretin.”

Reaction at 10 mM substrate was performed by supplying phloretin as an inclusion complex with 2‐hydroxypropyl‐β‐cyclodextrin (HPCD) for solubility enhancement (Li et al. 2023). The conversion of phloretin is shown in Supporting Information S1: Figure 14a, that of the sugar nucleotides in Supporting Information S1: Figure 14b. Release of the intermediary 3'‐*C*‐β‐xylosyl phloretin was not complete after 24 h (∼80%). Despite 10‐times more *Fc*CGT used compared to the 1.0 mM reaction (Figure 4b), the initial rate of *C*‐β‐xylosylation was only about doubled (Supporting Information S1: Table S3) and the βXyl phloretin formation was linear with time up to 2 h (Supporting Information S1: Figure 14). UDP‐xylose accumulated to slightly over ∼2 mM within the first 2 h and to almost ∼6 mM after 24 h (Supporting Information S1:Figure 14b), indicating that the donor substrate supply from UDP‐glucuronic acid was not limiting. However, the free phloretin released from the HPCD complex at a steady state might be too low to saturate the *Fc*CGT and so limit the apparent enzyme activity.

To enhance the *Fc*CGT activity for an accelerated *C*‐β‐xylosylation of phloretin, we considered a temperature increase from the standard 30°C to 40°C. We additionally considered in situ supply of UDP‐glucose through the reaction of sucrose synthase, sucrose + UDP ↔ UDP‐glucose + fructose (Figure 4a). The UDP released in the first *C*‐glycosylation step would thus be recycled into sugar nucleotide in the form of UDP‐glucose. The rate of βXyl phloretin formation at 40°C was increased 2.3‐fold (Supporting Information S1: Table 3) and the conversion of phloretin was complete within 24 h (Figure 4d). UDP‐xylose increased gradually to ∼7 mM during the first *C*‐glycosylation (Figure 4e). The *C*‐β‐glucosylation in the second step proceeded efficiently (Figure 4d) to give the desired βGlc‐βXyl phloretin at complete conversion. The UDP‐glucose was approximately constant at ∼6 mM (Figure 4e), indicating the proper function of the sucrose synthase under the conditions used. The UDP increased slightly (3.5–5.0 mM) in ways not fully accounted for by the mass balance for the enzymatic reactions. The full description of the usage of sugar nucleotides in the di‐*C*‐glycosylation process is shown in Figure 4e. The concentrations of UDP‐glucuronic acid and UDP‐xylose were constant (ratio of ∼0.38 = 2.5 mM/6.5 mM) during the second step of *C*‐glycosylation. Isolation of the βGlc‐βXyl phloretin from the reaction mixture with HPCD should be possible by applying methodology from earlier work on the synthesis of the 3',5'‐di‐*C*‐β‐glucoside of phloretin (Li et al. 2023). However, this was not pursued in the current study.

In conclusion, this study presents an in‐depth characterization of the hetero‐di‐*C*‐glycosylation of the polyphenol phloretin by *Fc*CGT. We show instalment of the βGal‐βGlc and βGlc‐βXyl structural motifs in excellent selectivity to obtain a unique di‐*C*‐glycosyl product in each case. The transformations proceed in complete yield based on the limiting concentration of phloretin used. Reaction time course studies provide detailed kinetic and thermodynamic insight into the one‐pot, two‐step *C*‐glycosylation process. The hetero‐di‐*C*‐glycosyl compounds were isolated in high purity. Integration of the di‐*C*‐glycosylation into a cascade transformation that provides the sugar nucleotide donors in situ was shown for practical synthesis of βGlc‐βXyl phloretin. The product was obtained at ∼6 g/L (10 mM) in quantitative conversion. The results showcase a feasible method for the intensified production of hetero‐di‐*C*‐glycosyl polyphenols. Facilitated access to these rare glycoside natural products promotes study of their function and enables exploration of their possible uses. Stability of the di‐*C*‐glycosylated polyphenols during production (e.g., under agitated conditions, see Li et al. 2023) might still constitute a challenging task to be addressed by biochemical engineering for scale‐up.

## 1 Methods

### 1.1 Encapsulation of Phloretin by HPCD

HPCD inclusion complexes of phloretin were prepared according to a literature protocol (Li et al. 2023), with further details given in the Supporting Information.

### 1.2 Enzyme Production

Expression and purification of *Fc*CGT, *Gm*SuSy and hUXS1 were described in the literature (Eixelsberger et al. 2012; Li et al. 2023), with further details given in the Supporting Information.

### 1.3 Enzyme Activity Assays

The *C*‐glycosylation activity of *Fc*CGT towards phloretin and phloretin mono‐*C*‐glycosides was determined from the initial phase of the first or second step in the *Fc*CGT reactions (1.0 mM acceptor substrate, 2.0 mM donor substrate). The initial formation rates of mono‐*C*‐glycosides and hetero‐di‐*C*‐glycosides were calculated from the corresponding time courses (Figures 2b, 3b; Supporting Information S1: Figures 7a, 12a, and 15a). The activity of hUXS1 on UDP‐GlcA (2.0 mM) was determined from a time course (Supporting Information S1: Figure 16) of the reactions with 0.5 mM NAD^+^. Full details of the activity assays are provided in the Supporting Information under “Enzyme activity assays”.

### 1.4 Determination of Kinetic Parameters

Reactions were performed at a varied concentration of the sugar donor (UDP‐Glc, UDP‐Gal or UDP‐Xyl) from 20 to 800 μM and a constant saturating concentration of phloretin (1.0 mM) at pH 8.0 and 30°C in a total volume of 100 μL. *Fc*CGT was used at a concentration (0.005–1.0 mg/mL) adjusted to compensate the differences in enzyme activity with the different sugar nucleotides. After incubating the mixture for a suitable time, typically up to 20 min, the reaction mixtures were quenched with MeOH and centrifuged at 21130 g for 20 min. The cleared supernatants were analyzed by HPLC. All experiments were performed twice and the results were in good agreement. Reaction rates were calculated as [UDP] formation/time elapsed from linear parts of the related time courses. The values of *K*_M_ and *k*_cat_ were determined from hyperbolic Michaelis–Menten plots using nonlinear least‐squares fitting with Origin 2021 (Supporting Information S1: Figure 17). The *k*_cat_ was calculated from the maximum rate (*V*_max_) with the molar enzyme concentration [E], using the relationship *k*_cat_ = *V*_max_/[E]. [E] was obtained from mass‐based protein concentration and the molecular weight of the *Fc*CGT polypeptide (*M*_*r*_ = 53600).

### 1.5 Cascade Reactions for the Synthesis of βGlc‐βXyl Phloretin

#### 1.5.1 *Fc*CGT/hUXS1 cascade reactions

The reactions were performed in 50 mM potassium phosphate (pH 8.0) buffer containing phloretin (1.0 mM; 2% DMSO as cosolvent), NAD^+^ (2.0 mM), 2‐mercaptoethanol (10 mM), *Fc*CGT (0.50 mg/mL) and hUXS1 (1.5 mg/mL) in the final volume of 100 μL. The reactions were initiated by the addition of UDP‐GlcA (2.0 mM) and carried out at 30°C. The sampling and HPLC analysis were carried out as described in the Supporting Information under “Enzyme activity assays”. After phloretin was fully converted, an additional 2.0 mM UDP‐Glc (final concentration) was added to start the second step.

#### 1.5.2 *Fc*CGT/hUXS1/*Gm*SuSy cascade reactions

The reactions were performed in 50 mM potassium phosphate (pH 8.0) buffer containing HPCD‐encapsulated phloretin (10 mM), NAD^+^ (2.0 mM), 2‐mercaptoethanol (10 mM), *Fc*CGT (5.0 mg/mL), and hUXS1 (2.5 mg/mL) in the final volume of 100 μL. The reactions were initiated by the addition of UDP‐GlcA (20 mM) and carried out at 30°C or 40°C without agitation. For reactions at 40°C, 1.0 mg/mL *Gm*SuSy and 100 mM sucrose (final concentration) were added to start the second step, after phloretin was completely converted based on HPLC analysis.

### 1.6 Hetero‐di‐*C*‐Glycoside Production and Isolation

The preparative reactions were performed using *Fc*CGT (0.50–5.0 mg/mL) and contained 2.0 mM phloretin. βGal‐βGlc phloretin was purified using silica column chromatography (HPLC purity ≥ 98%; 2.1 mg, 22% yield), while βGlc‐βXyl phloretin was isolated by preparative HPLC (HPLC purity ≥ 98%; 3.5 mg, 31% yield). The choice of these purification methods was somewhat arbitrary. Both can be used for product isolation but were not optimized for efficiency of recovery. Full details of the reactions and product isolations are provided in the Supporting Information under “Preparation and isolation of hetero‐di‐*C*‐glycoside”.

### 1.7 Analytical Methods

#### 1.7.1 HPLC

UDP‐sugars and UDP were separated using a Shimadzu Prominence HPLC‐UV system (Shimadzu, Korneuburg, Austria) on a Kinetex C18 column (5 µm, 100 Å, 50/250 × 4.6 mm) with an isocratic method. The mobile phase consisted of acetonitrile (or methanol) and tetrabutylammonium bromide (TBAB) buffer (40 mM TBAB, 20 mM K₂HPO₄/KH₂PO₄, pH 5.9). Detection of UDP‐sugars and UDP was performed by UV at 262 nm. Acceptor substrates and glycoside products were separated on an Agilent 1200 Series HPLC‐UV system (Morges, Switzerland) using a Kinetex C18 column (3 µm, 100 Å, 200 × 4.6 mm) with a gradient method (0.6 mL/min) of water and acetonitrile (each containing 0.1% formic acid, v/v) as the mobile phase. The gradient method was as follows: 0–10 min, linear, 20% to 75% MeCN; 10–11 min, isocratic, 75% MeCN; 11–11.01 min, linear, 75% to 20% MeCN; 11.01–13 min, isocratic, 20% MeCN. Detection of the acceptor substrates and glycoside products was carried out at 288 nm. The details of the HPLC methods used in the analysis of UDP‐sugars and UDP are given in Supporting Information S1: Table 4. The methods of HPLC‐MS used in the analysis of hetero‐di‐*C*‐glycosides are provided in the Supporting Information under “HPLC‐UV/MS”. The relative ratios of each component in reactions were determined based on the proportion of each peak area to the total peak area of all components in HPLC. The concentrations of phloretin/glycosides or UDP‐sugars/UDP were calculated by multiplying the initial concentration of phloretin or UDP‐sugar by their relative ratios.

#### 1.7.2 NMR

The identity of the synthesized hetero‐di‐*C*‐glycosides was determined by ^1^H and ^13^C NMR. Full details of the methods are provided in the Supporting Information under “NMR analysis of phloretin hetero‐di‐*C*‐glycosides”.

## Author Contributions

**Tuo Li**, **Annika J. E. Borg**, and **Bernd Nidetzky**, design of study. **Tuo Li**, enzymatic reactions. **Annika J. E. Borg**, **Leo Krammer**, and **Tuo Li**, product isolation and interpretation of NMR spectra of hetero‐di‐*C*‐glycoside products. **Rolf Breinbauer**, supervision and resources. **All authors**, discussion and manuscript editing. **Tuo Li**, **Annika J. E. Borg**, **Bernd Nidetzky**, manuscript draft. **Tuo Li**, **Bernd Nidetzky**, final manuscript. **Bernd Nidetzky**, supervision and funding acquisition.

## Conflicts of Interest

The authors declare no conflicts of interest.

## Supporting information

Supporting information.

## Data Availability

The data that support the findings of this study are available from the corresponding author upon reasonable request. The data that support the findings of this study are available in the supporting material of this article and from the corresponding author upon reasonable request.
