# Supplementary material for: One‐Pot Hetero‐Di‐C‐Glycosylation of the Natural Polyphenol Phloretin by a Single C‐Glycosyltransferase With Broad Sugar Substrate Specificity
Source: Biotechnol Bioeng. 2025 Feb 7;122(5):1296–304. doi: 10.1002/bit.28948 (PMC11975207; doi:10.1002/bit.28948)
Supplement: Supplementary file 1 — Supporting information. [file BIT-122-1296-s001.pdf]

Supporting Information

One-pot hetero-di-*C*-glycosylation of the natural polyphenol phloretin by a single *C*-glycosyltransferase with broad sugar substrate specificity

Tuo Li[1], Annika J. E. Borg[1,2], Leo Krammer[3], Rolf Breinbauer[3] and Bernd Nidetzky[1,2*]

[1] Institute of Biotechnology and Biochemical Engineering, Graz University of Technology, NAWI Graz, Petersgasse 12/1, 8010 Graz, Austria

[2] Austrian Centre of Industrial Biotechnology (acib), Krenngasse 37, 8010 Graz, Austria

[3] Institute of Organic Chemistry, Graz University of Technology, NAWI Graz, Stremayrgasse 9, 8010 Graz, Austria

* Corresponding author; e-mail: bernd.nidetzky@tugraz.at

Materials and methods – addition to main text

Materials

Phloretin (>98%), HPCD (>96%), UDP (>97%) and UDP-Glc (>98%) were from Carbosynth (Compton, UK). UDP-Gal (>95%), and UDP-GlcA (>98%) were from Sigma Aldrich (Vienna, Austria). Sucrose (>99%), and 2-mercaptoethanol (>99%) were from Roth (Karlsruhe, Germany). Nothofagin (3'-*C*-β-glucosyl phloretin; >99%) was obtained from a previous study using enzymatic synthesis (Schmölzer et al., 2018). UDP-Xyl (>99%) was obtained from a previous study using enzymatic synthesis (Savino et al., 2019). All other reagents and chemicals were of highest available purity and purchased from Sigma Aldrich/Fluka (Vienna, Austria), Roth (Karlsruhe, Germany) or Merck (Vienna, Austria).

Encapsulation of phloretin by HPCD (2-hydroxypropyl-β-cyclodextrin)

First, HPCD (1.05 g, 0.75 mmol) was dissolved in 1.0 mL of deionized water using microwave heating (Micro-Chef V98, Moulinex, Austria; 20 s at 750 W), followed by incubation in a drying chamber at 70 °C for 1 h. The Sarstedt tube was inverted every 15 min for thorough mixing. Next, phloretin (0.165 g, 0.6 mmol) was added and dissolved using the same method. After adjusting the final volume to 2.5 mL, the solution was centrifuged at 21130 g for 5 min at 22 °C to remove any insoluble phloretin.

Enzyme production

Briefly, the enzymes were overexpressed in *E. coli* BL21(DE3) (*Fc*CGT and *Gm*SuSy) or Rosetta 2(DE3) (hUXS1), using the respective expression vectors pET-28a (*Fc*CGT), pET-11a (hUXS1) or pETSTRP3 (*Gm*SuSy). Expression was carried out in Terrific Broth (TB) for *Fc*CGT and in Lysogeny Broth (LB) for *Gm*SuSy and hUXS1. His-tagged *Fc*CGT and hUXS1 were purified using a HisTrapTM HP column (5 mL resin, Cytiva, Uppsala, Sweden), while

Strep-tagged *Gm*SuSy was purified with a StrepTrapTM HP column (5 mL resin, Cytiva, Uppsala, Sweden). The size and purity of the enzymes were verified by SDS-PAGE. Following buffer exchange via Vivaspin ultrafiltration tubes (Sartorius, Goettingen, Germany), the proteins were stored at -20 °C.

Enzyme activity assays

Activity of *Fc*CGT: To assay the *C*-galactosylation activity toward phloretin, 1.0 mM phloretin (2% DMSO), 2.0 mM UDP-Gal and 1.0 mg/mL *Fc*CGT were used for the reactions. To assay the *C*-glucosylation activity toward βGal phloretin, 1.0 mM phloretin was first completely converted to βGal phloretin using 5.0 mg/mL *Fc*CGT, and 2.0 mM UDP-Gal. The second step was started by adding 2.0 mM UDP-Glc (final concentration), and used for measurement of *C*-glucosylation activity. For the *C*-xylosylation activity toward phloretin and the *C*-glucosylation activity toward βXyl phloretin, the reactions containing 1.0 mM phloretin (2% DMSO), 2.0 mM UDP-Xyl and 0.10 mg/mL *Fc*CGT were employed, and 2.0 mM UDP-Glc (final concentration) was added after the phloretin was fully converted to βXyl phloretin. The first and second step were used for the calculation of *C*-xylosylation, and *C*-glucosylation activity, respectively. The reactions containing 1.0 mM nothofagin (2% DMSO) and 2.0 mM UDP-Gal/UDP-Xyl were used for the assay of *C*-galactosylation activity (5.0 mg/mL *Fc*CGT) and *C*-xylosylation activity (0.50 mg/mL *Fc*CGT), respectively. To test the *Fc*CGT activity toward phloretin and UDP-GlcA, 1.0 mM phloretin (2% DMSO), 2.0 mM UDP-GlcA, 3.0 mg/mL *Fc*CGT were used for the reactions. All reactions (100 µL) were performed in potassium phosphate buffer (50 mM, pH 8.0) containing 10 mM 2-mercaptoethanol, and incubated at 30 °C without agitation. At desired time points, 5 µL of reaction mixture was quenched with methanol (90% (v/v) final concentration) and the precipitated enzyme removed by centrifugation (21130 *g*, 4 °C, 30 min) prior to HPLC analysis. The initial formation rates of

mono- and di-*C*-glycosides were calculated from the linear segments of their respective time courses. This was done by dividing the slope of the linear regression (mM/min) by the enzyme concentration (mg/mL), resulting in the initial rate expressed in µmol/(min·mg protein). One unit (U) of *Fc*CGT activity is defined as the amount of enzyme producing 1 µmol of product (mono-*C*-glycoside or hetero-di-*C*-glycoside) per minute under the specified conditions, where the acceptor substrate is in excess.

Activity of hUXS1: The reaction mixture (100 µL) contained 2.0 mM UDP-GlcA, 0.50 mM NAD^+ and 2.0 mg/L hUXS1 in potassium phosphate buffer (50 mM, pH 8.0). Reactions were performed at 30 °C and sampled as described for *Fc*CGT activity assays. The initial rate was determined from the linear part of the corresponding time course, by dividing the slope of the linear regression (mM/min) by the enzyme concentration (mg/mL), resulting in the initial rate expressed as µmol/(min·mg protein). One unit (U) of hUXS1 activity is defined as the amount of enzyme producing 1 µmol of UDP-Xyl from UDP-GlcA per minute under the specified conditions, where UDP-GlcA is present in excess.

Preparation and isolation of hetero-di-*C*-glycoside

βGal-βGlc phloretin: The reactions (8 × 1.0 mL) were carried out in 2 mL Eppendorf tubes under the following reaction conditions: 2.0 mM phloretin (4% DMSO as co-solvent), 5.0 mM UDP-Gal, 5.0 mg/mL *Fc*CGT, and 10 mM 2-mercaptoethanol in potassium phosphate buffer (50 mM, pH 8.0) at 30 °C. After 48 h, 10 µL of reaction mixture were quenched and subjected to HPLC analysis as described in the Supporting Information under “Enzyme activity assays”, to make sure that phloretin was fully converted to the corresponding *C*-galactoside. UDP-Glc (4.0 mM, final concentration) were added to start the second step, and another 10 µL of reaction mixture were sampled as described above after 12 h (full conversion of intermediate to βGal-βGlc phloretin). The whole reaction was quenched by removing the enzymes using Vivaspin

20 ultrafiltration tubes (10,000 MWCO PES; Sartorius, Goettingen, Germany), at 3220 g and 4 °C. Subsequently, solvent in the reaction mixture was removed under reduced pressure on a Laborota 4000 rotary evaporator (Heidolph, Schwabach, Germany) at 40 °C. The crude product was resuspended in 2.0 mL solvent mixture of 1-butanol, acetic acid, and deionized water (2:1:1), and loaded into a silica 60 column (20 mL, 0.04-0.063 mm, Machery-nagel, Duren, Germany) for purification of βGal-βGlc phloretin. The column was washed with the solvent mixture, and the eluted fractions analyzed on HPLC. The product-containing fractions were pooled and the solvent removed by rotary evaporator (as described above) and lyophilization (Christ Alpha 1-4 lyophilizer, B. Braun Biotech International, Melsungen, Germany). The identity of βGal-βGlc phloretin was analyzed on NMR.

βGlc-βXyl phloretin: The reactions (10 × 1.0 mL) were carried out in 2 mL Eppendorf tubes under the following reaction conditions: 2.0 mM phloretin (4% DMSO as co-solvent), 4.0 mM UDP-GlcA, 0.50 mg/mL *Fc*CGT, 4.0 mM NAD^+, 1.5 mg/mL hUXS1, and 10 mM 2-mercaptoethanol in potassium phosphate buffer (50 mM, pH 8.0) at 30 °C. When phloretin was fully converted to the corresponding *C*-xyloside (after 10 h of reaction) based on HPLC analysis, 4.0 mM UDP-Glc (final concentration) were added to start the *C*-glucosylation of the intermediate. When βXyl phloretin was fully converted to βGlc-βXyl phloretin (after 2 h of reaction), the whole reaction mixtures were quenched, filtered, and the solvent was removed as described under "βGal-βGlc phloretin". The product mixture was re-dissolved in 0.50 mL of deionized water, and subjected to preparative HPLC for product isolation using the method described under "Reversed-Phase Preparative HPLC". The identity of βGlc-βXyl phloretin was confirmed on NMR.

Reversed-Phase Preparative HPLC

Reversed-phase preparative HPLC purifications were performed on a Thermo Scientific UltiMate 3000 system. Detection was accomplished with a Dionex UltiMate Diode Array Detector. The separations were carried out on a Macherey Nagel 125/21 Nucleodur® 100-5 C18EC (125 × 21 mm, 5 µm) column. Acetonitrile and water with 0.1% trifluoroacetic acid were used as eluents for the purification of the compounds. Typically, the product fractions obtained from preparative HPLC were concentrated under reduced pressure to remove the organic solvents and then lyophilized to obtain the product. The following method was applied: Method_Prep: T = 30 °C, constant flow rate: 15 mL/min; 0.0-3.0 min, isocratic, 2% MeCN (98% H_2O + 0.1% TFA), 3.0-16.0 min, linear, 2% to 50% MeCN (98% to 50% H_2O + 0.1% TFA), 16.0-17.0 min, linear, 50% to 100% MeCN (50% to 0% H_2O + 0.1% TFA), 17.0-18.0 min, isocratic, 100% MeCN, 18.0-19.0 min, linear, 100% to 2% MeCN (0% to 98% H_2O + 0.1% TFA), 19.0-22.0 min, isocratic, 2% MeCN (98% H_2O + 0.1% TFA).

HPLC-UV/MS

Analytical HPLC-UV/MS measurements for βGlc-βXyl phloretin were performed on an Agilent Technologies 1200 Series system (G1379 Degasser, G1312 Binary Pump, G1367C HiP ALS SL Autosampler, G1330B FC/ALS Thermostat, G1316B TCC SL column compartment, G1365C MWD SL multiple wavelength detector (deuterium lamp, 190-400 nm)) equipped with a single quadrupole LCMS detector "6120 LC/MS" using electrospray ionization source (ESI in positive and negative mode). Separations were carried out on a C-18-Reversed-Phase column of the type "Poroshell® 120 SB-C18, 3.0 × 100 mm, 2.7 µm" by Agilent Technologies. Flow: Constant flow rate 0.7 mL/min, T = 35 °C. The following method was used: *MeCN_2_100*: 0.0-0.1 min, isocratic, 2% MeCN (98% H_2O + 0.05% TFA); 0.1-8.0 min, linear, 2% to 100% MeCN (98% to 0% H_2O + 0.05% TFA); 8.0-11.1 min, isocratic, 100%

MeCN; 11.1-11.3 min, linear, 100% to 2% MeCN (0% to 98% H_2O + 0.05 % TFA); 11.3-12.0 min, isocratic, 2% MeCN (98% H_2O + 0.05% TFA)

Analytical HPLC-UV/MS measurements for βGal-βGlc phloretin were performed on a "Shimadzu LCMS-2020" HPLC system with SCL-40 system controller, DGU-405 degassing unit, LC-40D XR solvent delivery module, SIL-40C XR auto sampler, SPD-40 UV-VIS detector, CTO-40C column oven, FCV-20AH2 valve unit and subsequent connected mass detector (Shimadzu LCMS-2020) with an electrospray ionization (ESI) source. Separations were carried out on a Waters ACQUITY UPLC CSH C18 column (130 Å, 1.7 µm, 2.1 mm × 50 mm, 1/pk). Signals were detected at 288 nm. As mobile phase acetonitrile (VWR HiPerSolv, HPLC-MS grade) and water (Barnstead NANOpure®, ultrapure water system) with 0.05 % formic acid (FA) were used. Flow: Constant flow rate 0.5 mL/min, T = 40 °C. The following method was used: 2-100% MeCN_H_2O 0.05% HCOOH: 0.0-0.2 min, isocratic, 2% MeCN (98% H_2O + 0.05% HCOOH); 0.2-6.5 min, linear, 2% to 100% MeCN (98% to 0% H_2O + 0.05% HCOOH); 6.5-7.9 min, isocratic, 100% MeCN; 7.9-8.5 min, linear, 100% to 2% MeCN (0% to 98% H_2O + 0.05 % HCOOH); 8.5-9.0 min, isocratic, 2% MeCN (98% H_2O + 0.05% HCOOH).

NMR analysis of phloretin hetero-di-*C*-glycosides

The identity of the synthesized hetero-di-*C*-glucosides were determined by ^1H NMR. The acquisitions of βGal-βGlc phloretin were carried out in deuterated methanol (Methanol-d_4, 99.80% ^2H, Eurisotop, Saint-Aubin Cedex, France) on a Jeol JNM-ECZL 400 MHz NMR Spectrometer with Royal HFX-Probe (autosampler with automatic tuning and matching). The acquisitions of βGlc-βXyl phloretin were performed in deuterium oxide (D_2O, 99.96% ^2H, Euriso-Top, Saint-Aubin Cedex, France) on a Varian INOVA 500-MHz NMR spectrometer (Agilent Technologies, Santa Clara, California, USA). The spectra were analyzed using

MestReNova 16.0. The products were identified based on their chemical shifts, by using reference values from literature (Zhang et al., 2020).

Table S1. Activities of *Fc*CGT toward different acceptors and donors.

Donor	Acceptor			
	Phloretin	Nothofagin	βGal phloretin	βXyl phloretin
UDP-Glc	3.02 U/mg (Li et al., 2023)	0.41 U/mg (Li et al., 2023)	5.05 ± 0.49 mU/mg	167 ± 1 mU/mg
UDP-Gal	4.60 ± 0.42 mU/mg	0.57 ± 0.05 mU/mg	ND†	/‡
UDP-Xyl	325 ± 21 mU/mg	26.3 ± 0.99 mU/mg	/‡	ND†

† "ND" means that no activity was detected.

‡ "/" means that the related experiments were not performed.

Table S2. Kinetic parameters of the *Fc*CGT toward different sugar donors.†

UDP-sugar	K_M (μM)	k_{cat} (min^{-1})	k_{cat}/K_M (min^{-1} $μM^{-1}$)
UDP-Glc	55 ± 2.4	139 ± 2.5	2.5
UDP-Gal	432 ± 26	0.28 ± 0.01	6.5×10^{-4}
UDP-Xyl	122 ± 11	15 ± 0.8	0.12

† Phloretin (1.0 mM) was used as a sugar acceptor. Michaelis-Menten plots are shown in Figure S17.

Table S3. Detailed information of the *Fc*CGT-hUXS1/*Gm*SuSy cascade reactions for the synthesis of βGlc-βXyl phloretin.

Entry†	Conc. of phloretin/ UDP-GlcA (mM)	Temp. (°C)	*Fc*CGT (mg/mL)	hUXS1 (mg/mL)	*Gm*SuSy (mg/mL)	Initial rate of βXyl formation (μM/min)‡	Duration of first step (h)	Initial rate of βGlc-β Xyl formation (μM/min)‡	Duration of second step (h)	Initial rate of UDP-GlcA consumption (μM/min)‡	Ref.
1	1.0 (phloretin)	30	0.10	/##	/##	32.5 ± 2.1	2.0	16.7 ± 0.1	2.0	/##	Fig. 3b, 3d
2	1.0/2.0	30	0.50	1.5	/##	7.7 ± 0.1	5.0	14.2 ± 0.1	3.0	17.1	Fig. 4b, 4c
3	10/20	30	5.0	2.5	/##	17.8 ± 0.2	/##	/##	/##	47.9	Fig. S14
4	10/20	40	5.0	2.5	1.0	41.7 ± 0.7	24	157 ± 2.5	1.5	123	Fig. 4d, 4e

† Entry 1, single *Fc*CGT for the first step; Entry 2-4, *Fc*CGT/hUXS1 cascade for the first step; Entry 1 and 3, single *Fc*CGT for the second step; Entry 4, *Fc*CGT/*Gm*SuSy cascade for the second step.

‡ The initial rates were determined from the linear part of the time courses by multiplying the slope of the linear regression (μM/min).

"/" means that it was not applicable under this circumstance.

Table S4. HPLC columns and methods used in the analysis of UDP-sugars and UDP. MeCN = acetonitrile, MeOH = methanol.

	HPLC columns	Methods
UDP-Gal/UDP-Xyl, and UDP	Kinetex C18 (5 µm, 100 Å, 50 × 4.6 mm)	Flow rate, 2.0 mL/min; 0-2 min, isocratic, 5% MeCN.
UDP-Gal, UDP-Glc and UDP	Kinetex C18 (5 µm, 100 Å, 250 × 4.6 mm)	Flow rate, 0.8 mL/min; 0-20 min, isocratic, 5% MeOH.
UDP-Xyl, UDP-Glc and UDP	Kinetex C18 (5 µm, 100 Å, 250 × 4.6 mm)	Flow rate, 0.8 mL/min; 0-20 min, isocratic, 10% MeOH.
UDP-GlcA, UDP-Xyl, and UDP	Kinetex C18 (5 µm, 100 Å, 50 × 4.6 mm)	Flow rate, 2.0 mL/min; 0-5 min, isocratic, 5% MeCN.
UDP-GlcA, UDP-Xyl, UDP-Glc and UDP	Kinetex C18 (5 µm, 100 Å, 250 × 4.6 mm)	Flow rate, 0.8 mL/min; 0-60 min, isocratic, 10% MeOH.

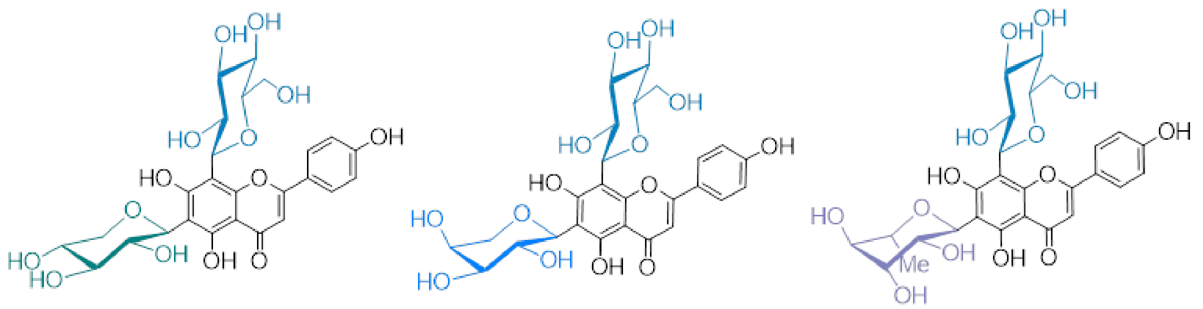

2b **3b** **4b**

Figure S1. Structure of compounds **2b** (vicenin-1), **3b** (isoschaftoside) and **4b** (isoviolanthin).

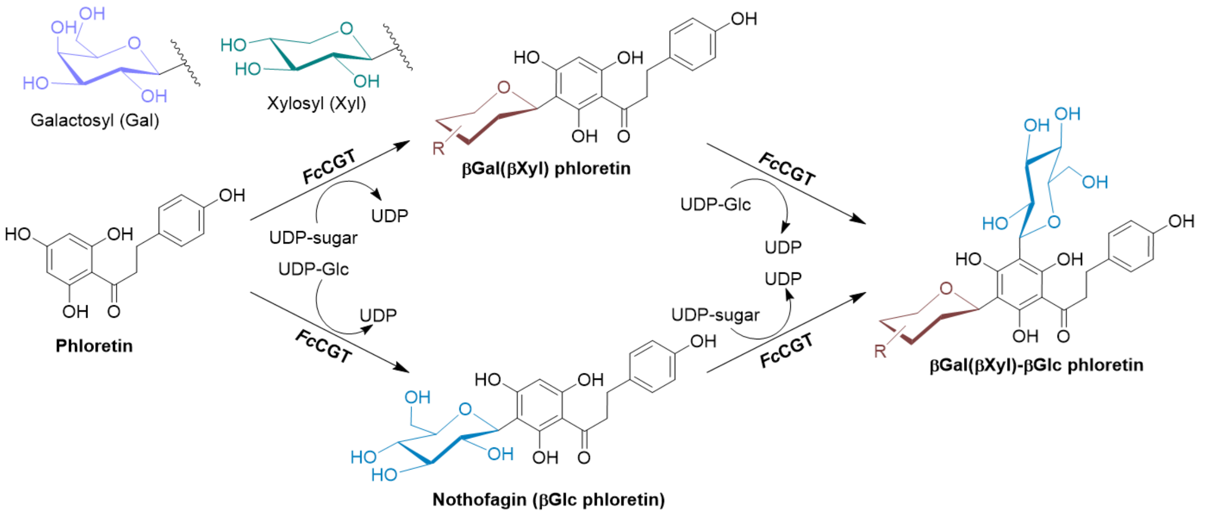

Figure S2. Reaction scheme for the synthesis of polyphenol hetero-di-*C*-β-glycosyl compounds through different routes. Upper route, *C*-glycosylation (*C*-galactosylation or C-xylosylation) followed by *C*-glucosylation, lower route, *C*-glucosylation followed by *C*-glycosylation (*C*-galactosylation or *C*-xylosylation).

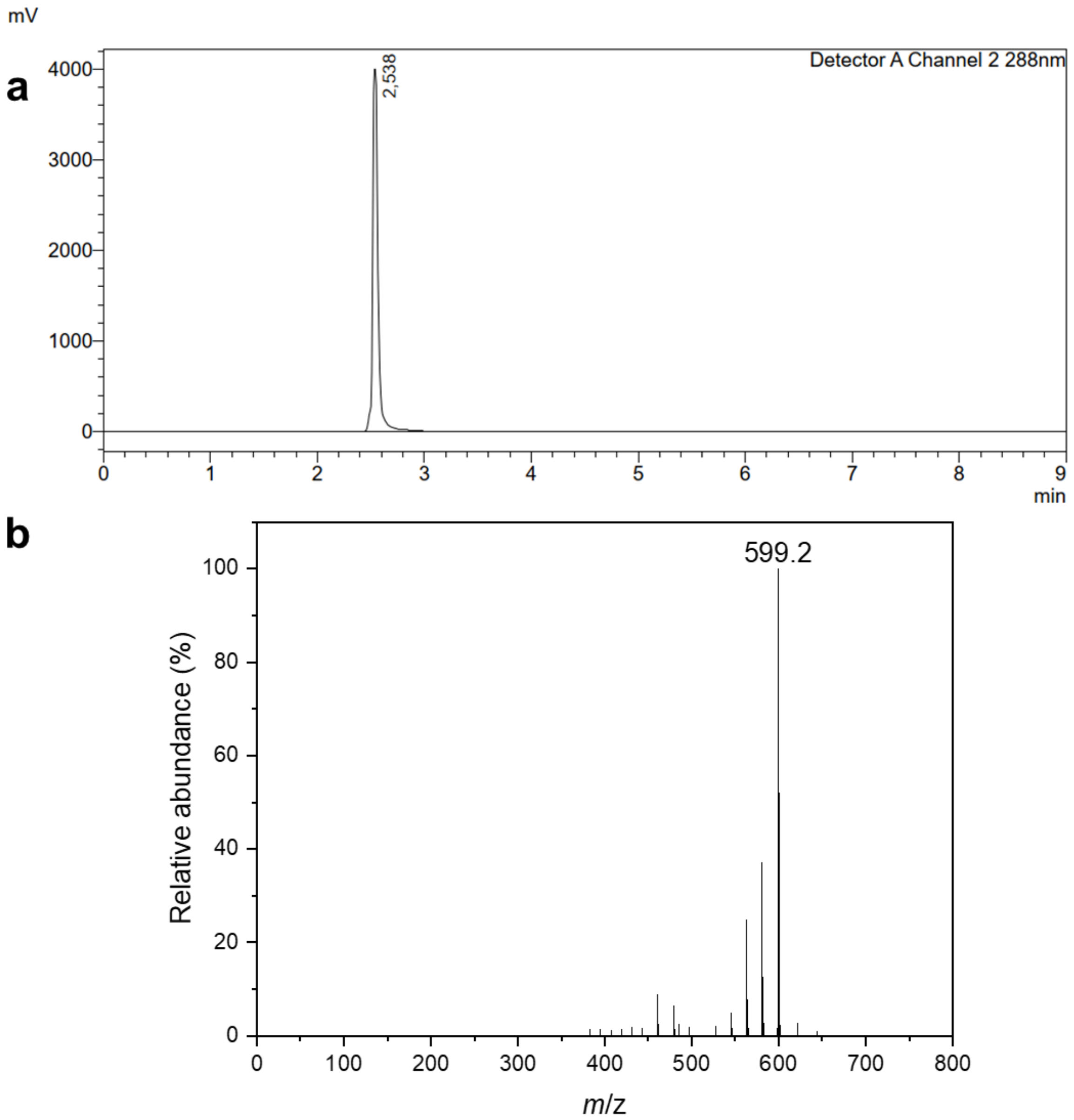

Figure S3. HPLC-UV/MS analysis of βGal-βGlc phloretin isolated by silica column chromatography. **a**. HPLC chromatogram of βGal-βGlc phloretin. **b**. Mass spectrum of βGal-βGlc phloretin corresponding to the HPLC chromatogram in (**a**); calculated $[M+H^+]^+ = 599.5$, found: 599.2.

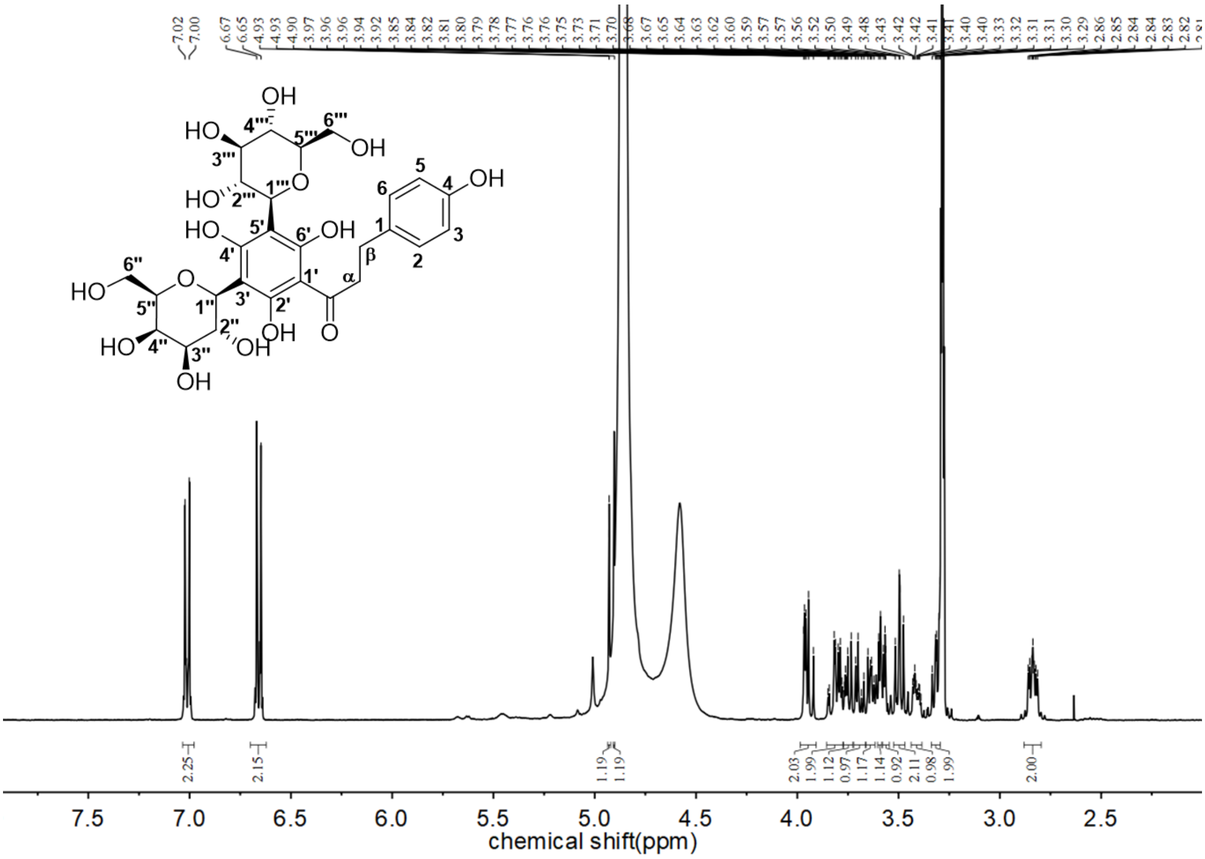

Figure S4. ^{1}H NMR spectrum (400 MHz, Methanol-d_4) of isolated βGal-βGlc phloretin. δ 7.02 (2H, d, *J* = 8.5 Hz, H-2, 6), 6.67 (2H, d, *J* = 8.5 Hz, H-3, 5), 4.95 - 4.93 (1H, m, H-1'''), 4.93 - 4.91 (1H, m, H-1''), 3.98 - 3.92 (2H, m, H-2'', 3'''), 3.87 - 3.78 (2H, m, H-6'''), 3.78 - 3.73 (1H, m, H_a-6''), 3.73 - 3.67 (1H, m, H_b-6''), 3.67 - 3.63 (1H, m, H-5''), 3.61 - 3.57 (2H, m, H-3'', 2'''), 3.53 - 3.48 (2H, m, H-4'', 4'''), 3.44 - 3.38 (1H, m, H-5'''), 3.35 - 3.31 (2H, m, H-α), 2.87 - 2.81 (2H, m, H-β).

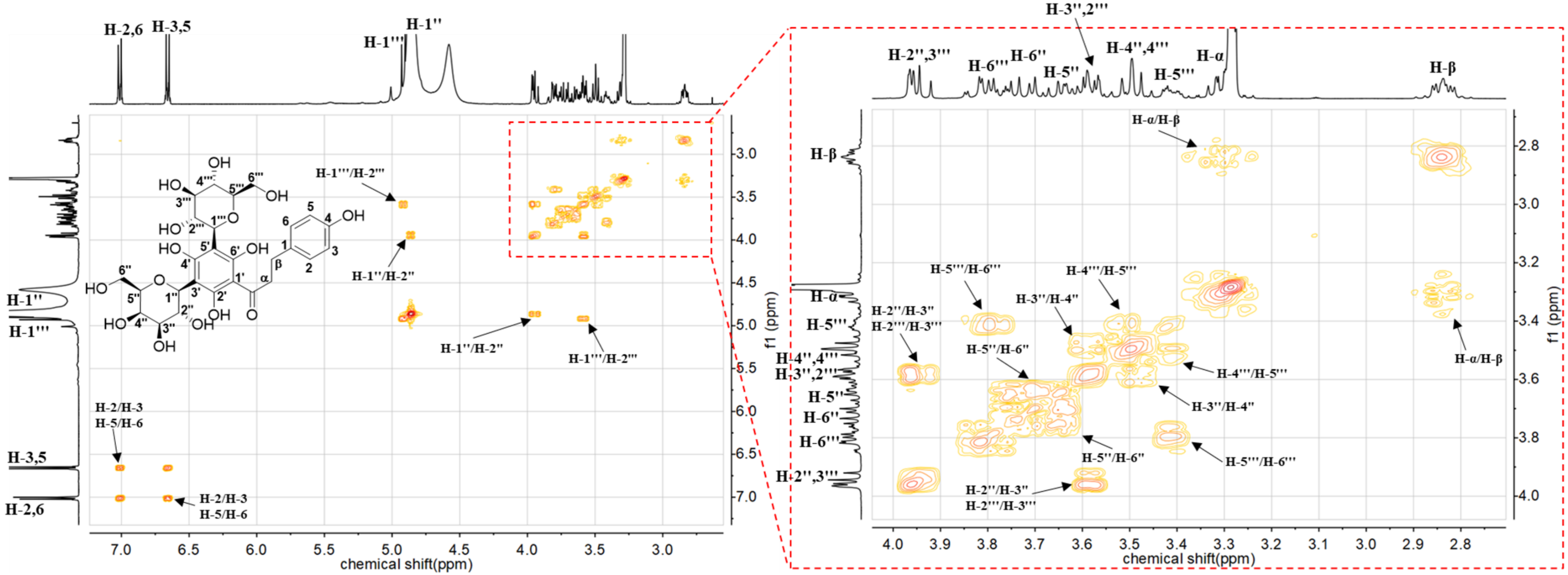

Figure S5. ^{1}H-^{1}H COSY spectrum (400 MHz, Methanol-d_4) of isolated βGal-βGlc phloretin.

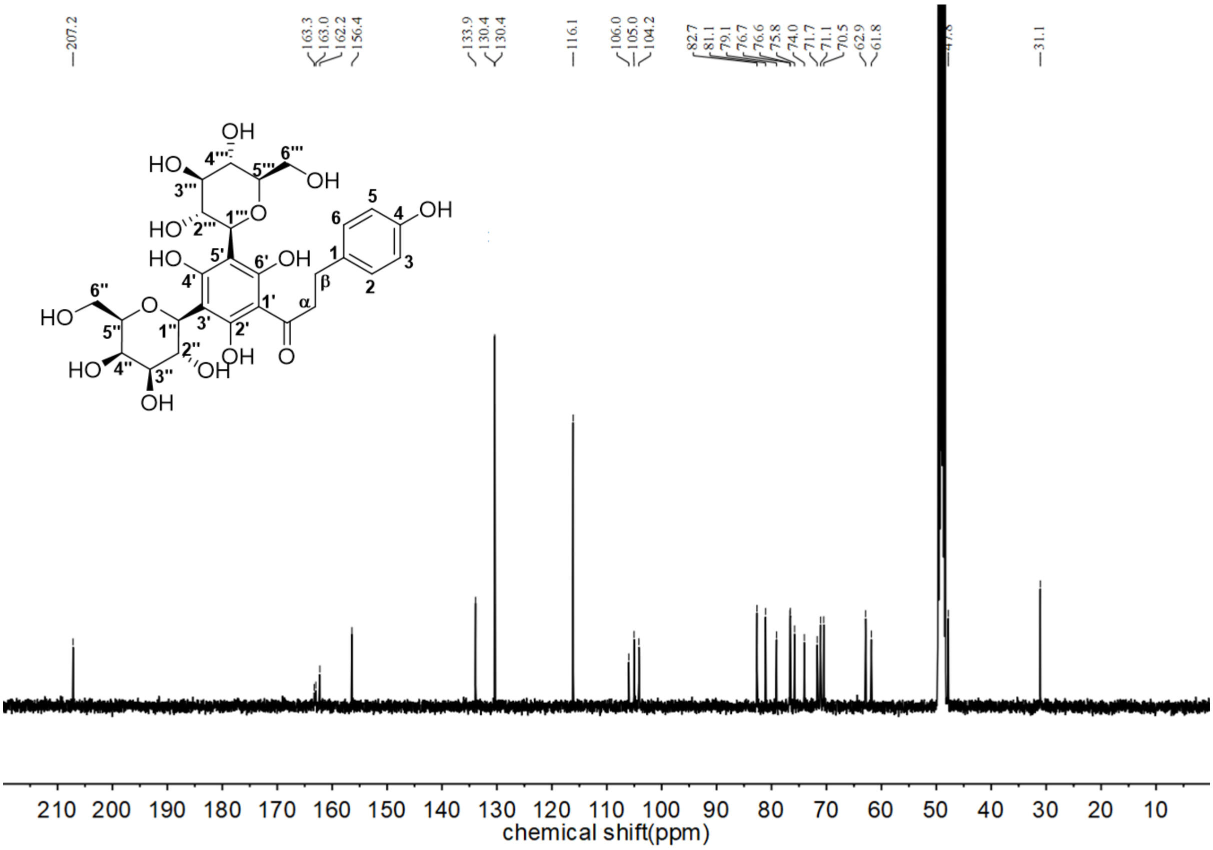

Figure S6. ^{13}C NMR spectrum (101 MHz, Methanol-d_4) of isolated βGal-βGlc phloretin. δ 207.2 (C=O), 163.3 (C-2'), 163.0 (C-6'), 162.2 (C-4'), 156.4 (C-4), 133.9 (C-1), 130.4 (C-2, 6), 116.1 (C-3, 5), 106.0 (C-1'), 105.0 (C-3'), 104.2 (C-5'), 82.7 (C-5'''), 81.1 (C-5''), 79.1 (C-3'''), 76.7 (C-1'''), 76.6 (C-1''), 75.8 (C-3''), 74.0 (C-2'''), 71.7 (C-2''), 71.1 (C-4'''), 70.5 (C-4''), 62.9 (C-6''), 61.8 (C-6'''), 47.8 (C-α), 31.1 (C-β).

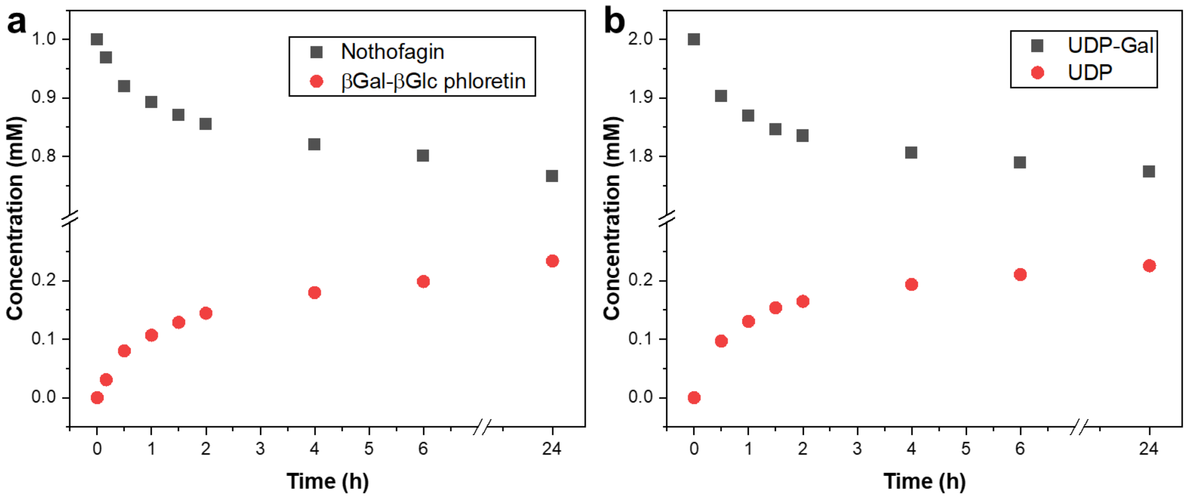

Figure S7. Time courses of nothofagin/βGal-βGlc phloretin (**a**) and UDP-Gal/UDP (**b**) in *Fc*CGT reactions used for measuring the *C*-galactosylation activity of *Fc*CGT with nothofagin. Reactions (100 µL) contained 1.0 mM nothofagin, 2.0 mM UDP-Gal, 5.0 mg/mL *Fc*CGT.

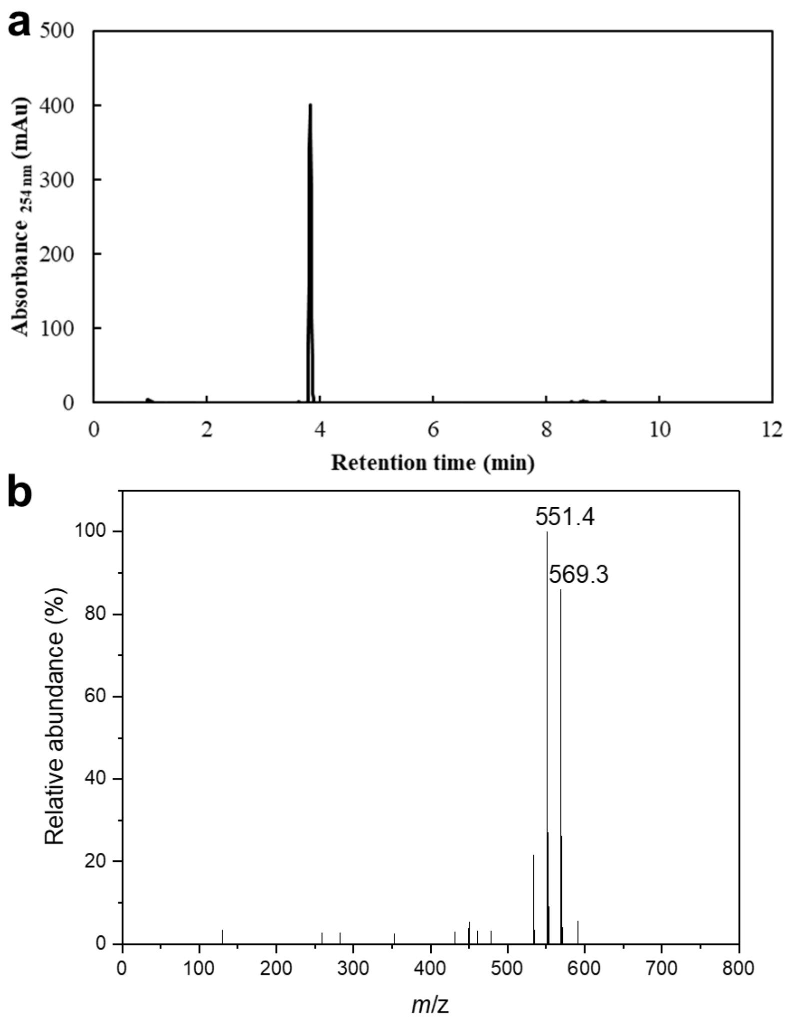

Figure S8. HPLC-UV/MS analysis of βGlc-βXyl phloretin isolated by prep-HPLC. **a**. HPLC chromatogram of βGlc-βXyl phloretin. **b**. Mass spectrum of βGlc-βXyl phloretin corresponding to the HPLC chromatogram in (**a**); calculated $[M+H^+]^+$ = 569.5, found: 569.3.

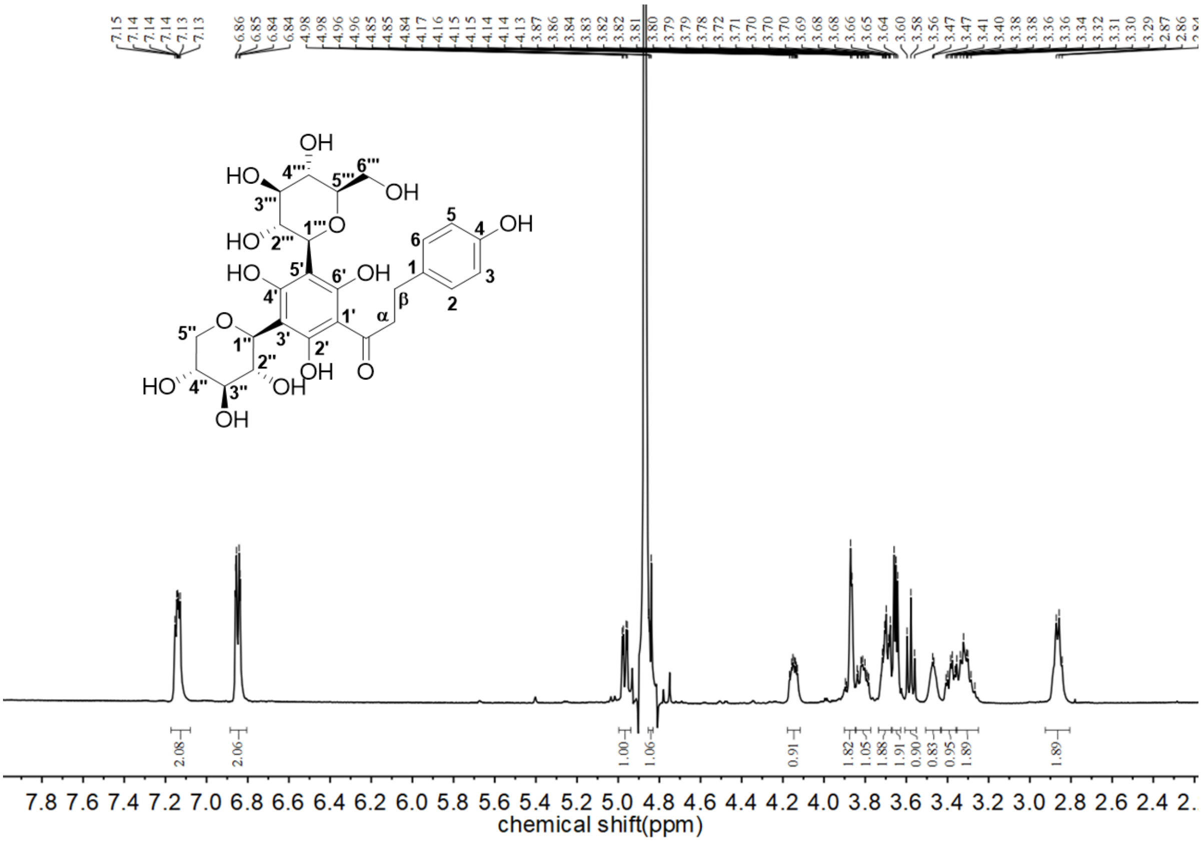

Figure S9. ^{1}H NMR spectrum (500 MHz, D_2O) of isolated βGlc-βXyl phloretin. δ 7.17 - 7.10 (2H, m, H-2, 6), 6.88 - 6.80 (2H, m, H-3, 5), 4.97 (1H, dd, J = 9.5, 2.4 Hz, H-1'''), 4.86 - 4.82 (1H, m, H-1''), 4.18 - 4.12 (1H, m, H_a-5''), 3.91 - 3.84 (2H, m, H-6'''), 3.85 - 3.78 (1H, m, H_b-5''), 3.74 - 3.67 (2H, m, H-2'', 2'''), 3.67 - 3.63 (2H, m, H-3''', 4'''), 3.58 (1H, t, J = 9.1 Hz, H-3''), 3.51 - 3.43 (1H, m, H-5'''), 3.39 (1H, dd, J = 11.1, 4.0 Hz, H-4''), 3.37 - 3.26 (2H, m, H-α), 2.91 - 2.82 (2H, m, H-β).

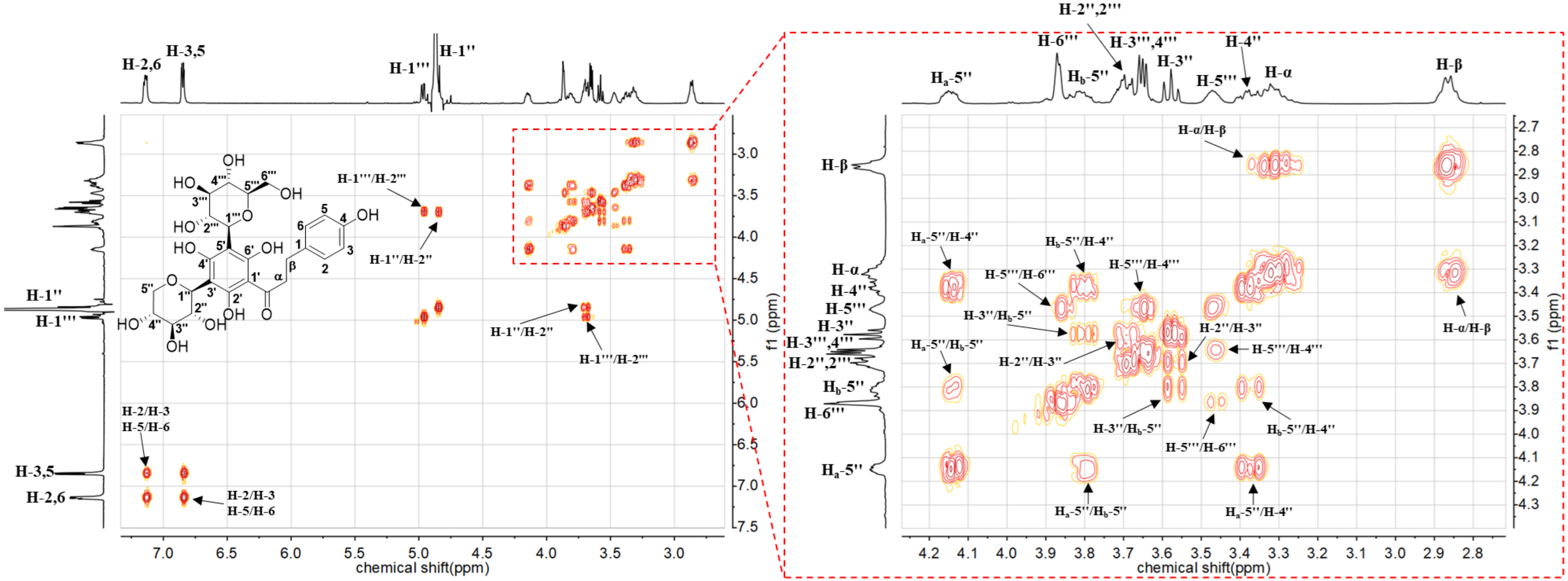

Figure S10. ^{1}H-^{1}H COSY spectrum (500 MHz, D_2O) of isolated βGlc-βXyl phloretin.

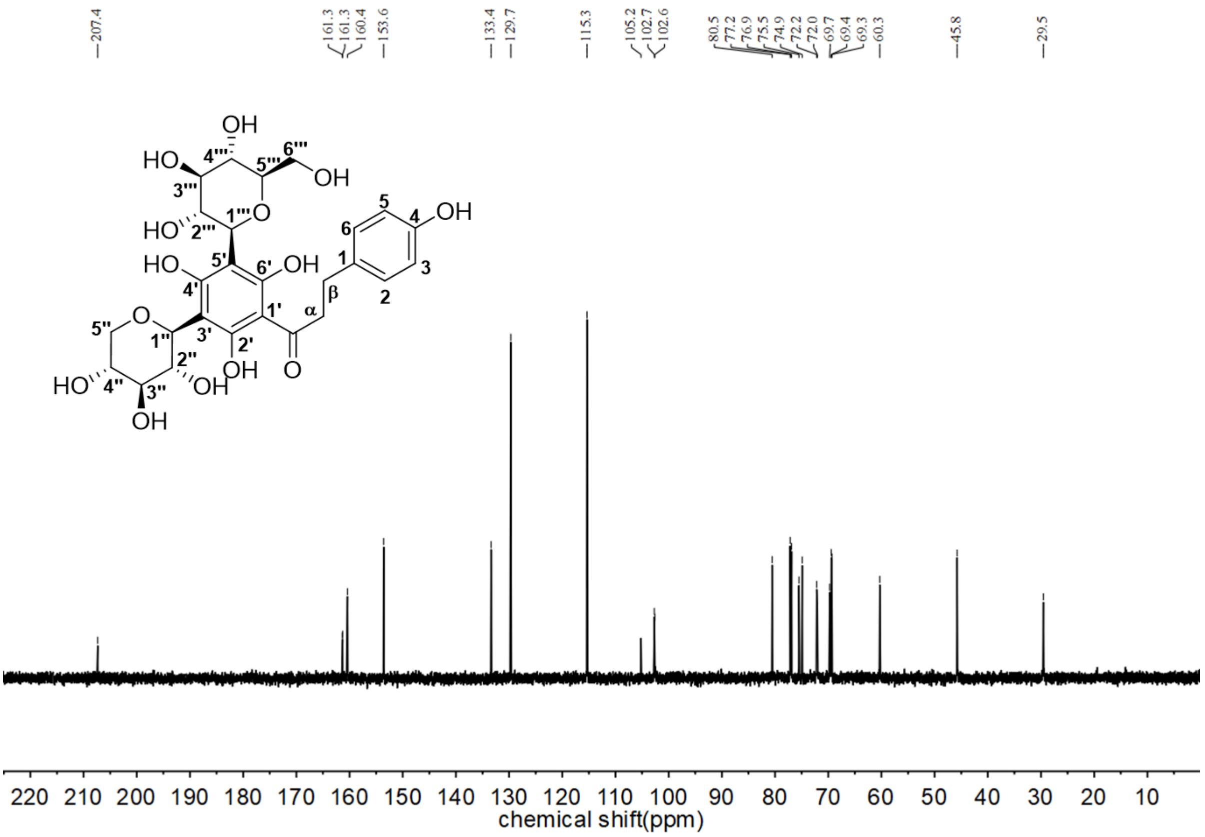

Figure S11. The ^{13}C NMR spectrum of isolated βGlc-βXyl phloretin in D_2O (126 MHz). δ 207.4 (C=O), 161.3 (C-2'), 161.3 (C-6'), 160.4 (C-4'), 153.6 (C-4), 133.4 (C-1), 129.7 (C-2, 6), 115.3 (C-3, 5), 105.2 (C-1'), 102.7 (C-3'), 102.6 (C-5'), 80.5 (C-5'''), 77.2 (C-3'''), 76.9 (C-3''), 75.5 (C-1''), 74.9 (C-1'''), 72.2 (C-2'''), 72.0 (C-2''), 69.7 (C-5''), 69.4 (C-4''), 69.3 (C-4'''), 60.3 (C-6'''), 45.8 (C-α), 29.5 (C-β).

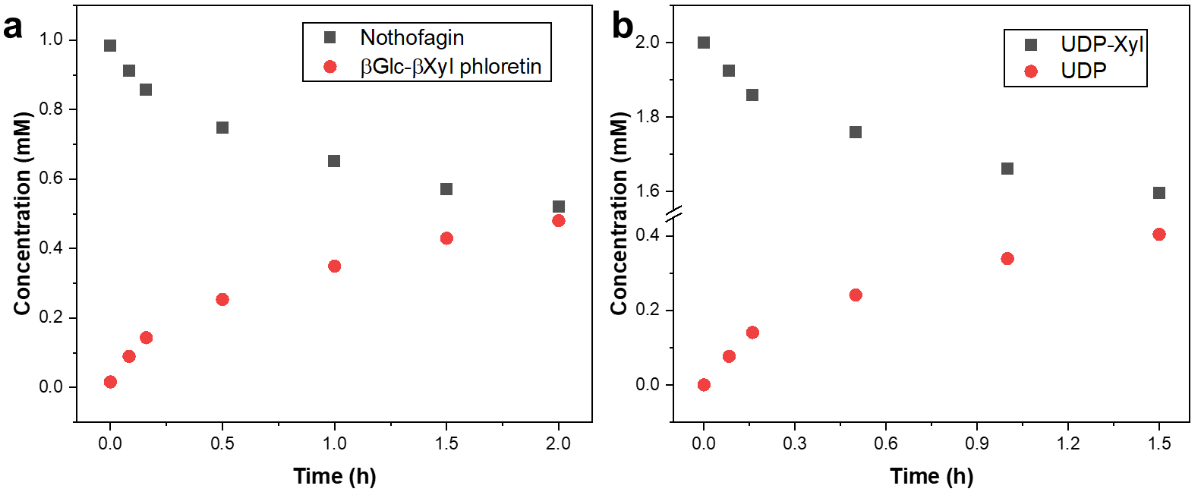

Figure S12. Time courses of nothofagin/βGlc-βXyl phloretin (**a**) and UDP-Xyl/UDP (**b**) in *Fc*CGT reactions used for measuring the *C*-xylosylation activity with nothofagin. Reactions (100 µL) contained 1.0 mM nothofagin, 2.0 mM UDP-Xyl, 0.50 mg/mL *Fc*CGT.

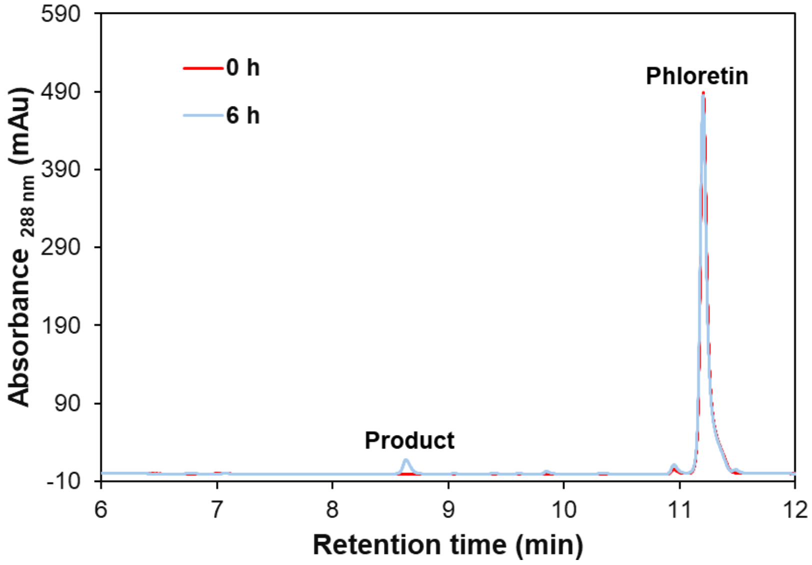

Figure S13. Overlay of HPLC chromatograms for *Fc*CGT/phloretin/UDP-GlcA reactions. Reactions (100 µL) contained 1.0 mM phloretin, 2.0 mM UDP-GlcA, 3.0 mg/mL *Fc*CGT, 10 mM 2-mercaptoethanol, in potassium phosphate buffer (50 mM, pH 8.0), and were carried out at 30 °C without agitation.

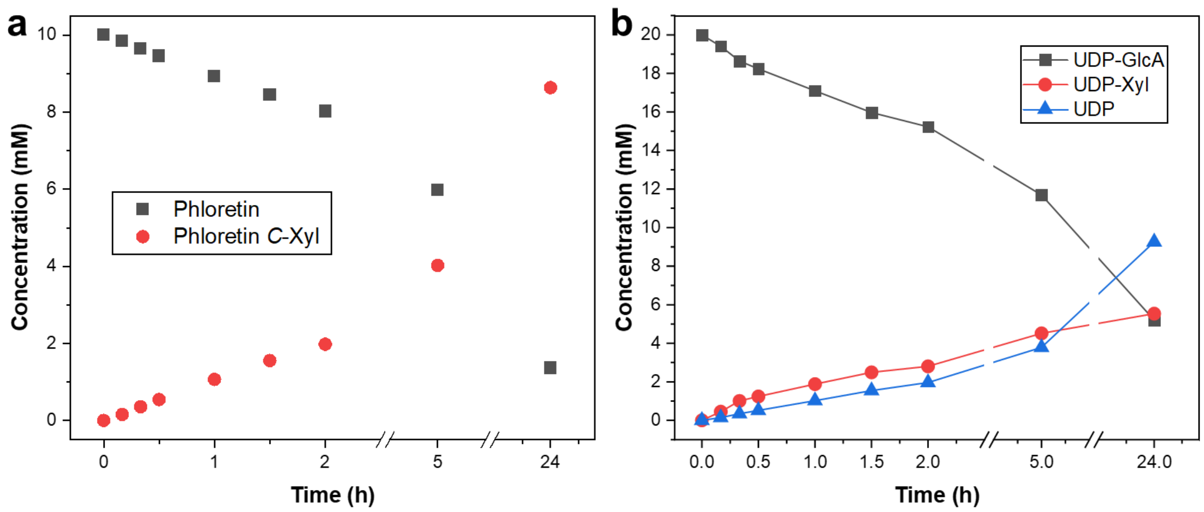

Figure S14. Time courses for phloretin/products (**a**), and UDP-sugar/UDP (**b**) in *Fc*CGT/hUXS1 cascade reactions (10 mM phloretin, 30 °C). Reactions (100 µL) contained 10 mM phloretin (HPCD-complex), 20 mM UDP-GlcA, 5.0 mg/mL *Fc*CGT, 2.5 mg/mL hUXS1, 2.0 mM NAD^+.

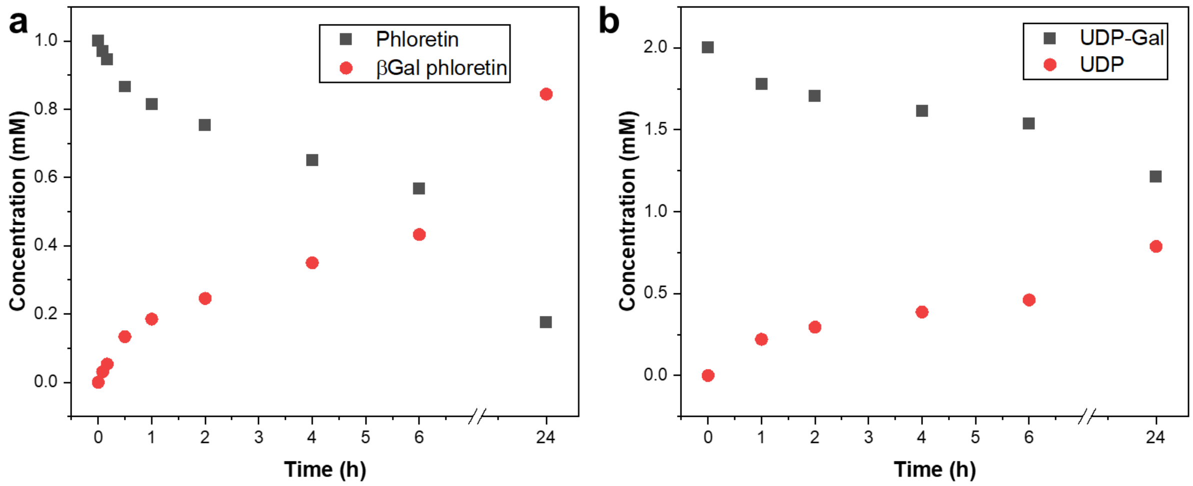

Figure S15. Time courses of phloretin/βGal phloretin (**a**) and UDP-Gal/UDP (**b**) in single *Fc*CGT reactions used for measuring the *C*-galactosylation activity of *Fc*CGT with phloretin. Reactions (100 μL) contained 1.0 mM phloretin, 2.0 mM UDP-Gal, 1.0 mg/mL *Fc*CGT.

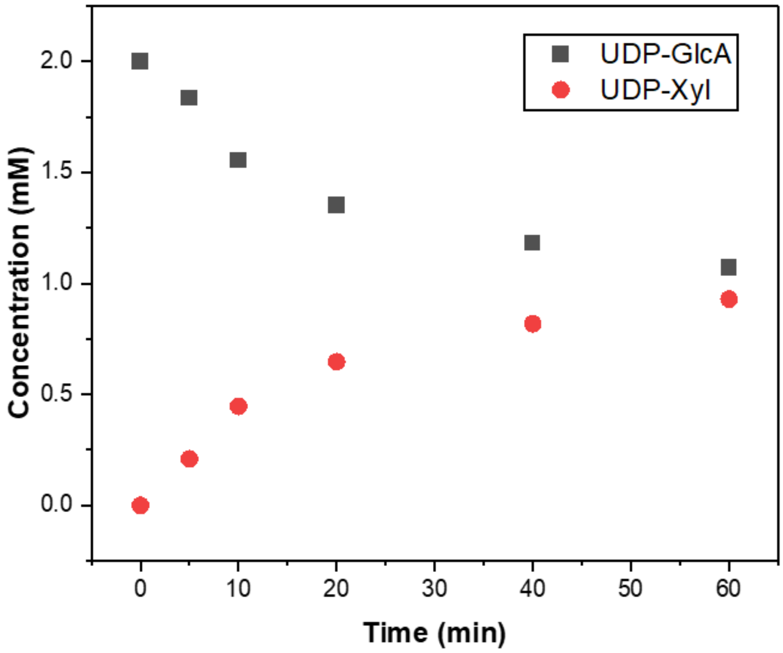

Figure S16. Time course in single hUXS1 reactions for the activity assay. Reactions (100 µL) contained 2.0 mM UDP-GlcA, 2.0 mM NAD^+, 2.0 mg/mL hUXS1, in potassium phosphate buffer (50 mM, pH 8.0), and were carried out at 30 °C without agitation. The activity of hUXS1 with UDP-GlcA (22 mU/mg) was calculated based on initial UDP-Xyl formation (0-10 min).

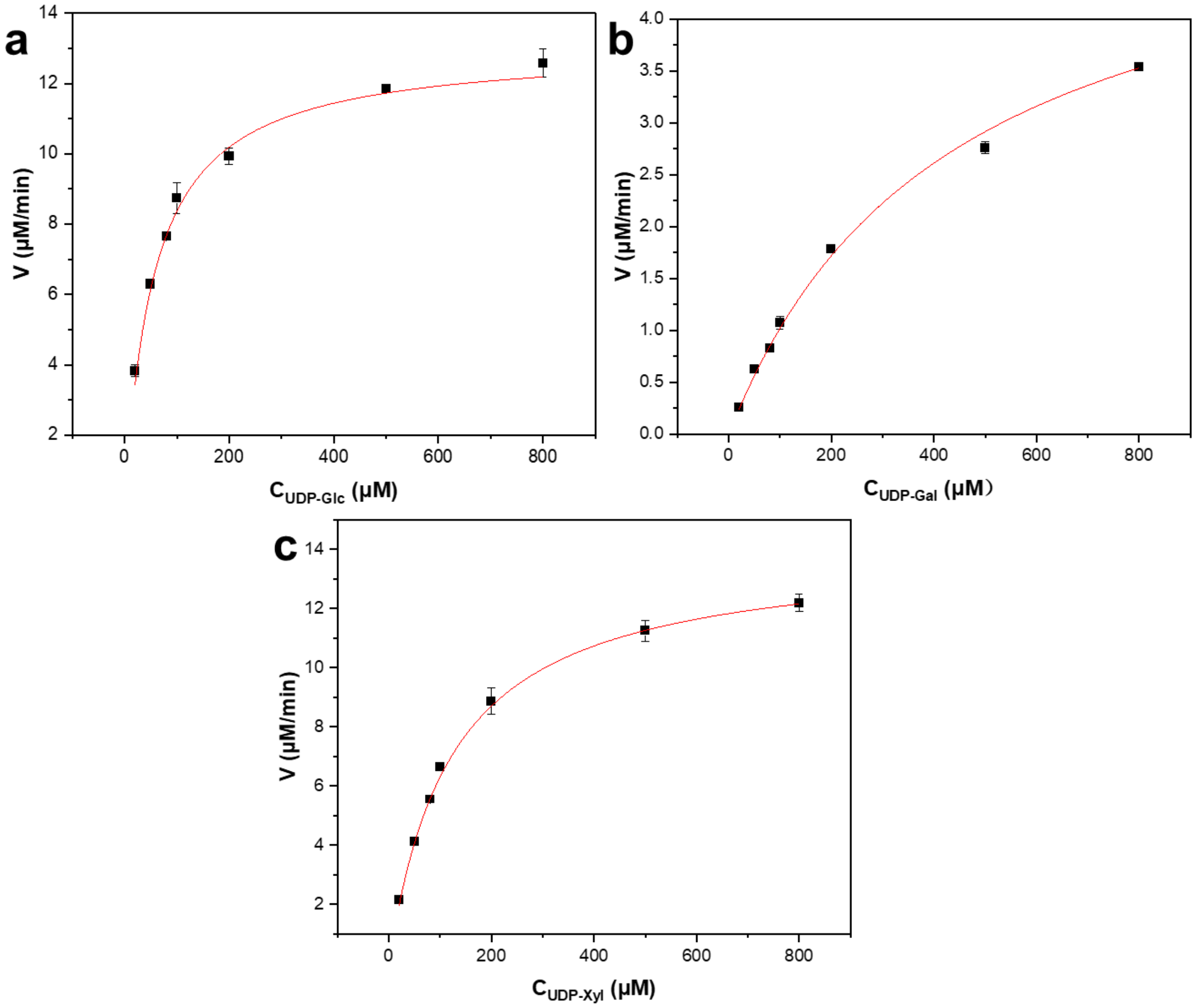

Figure S17. Michaelis-Menten plot for determination of kinetic parameters for *Fc*CGT toward UDP-Glc (**a**), UDP-Gal (**b**) and UDP-Xyl (**c**). Assays were performed in a final volume of 100 μL, consisting of 50 mM K_2HPO_4-KH_2PO_4 (pH 8.0), purified *Fc*CGT (0.093, 18.7 and 0.93 μM for UDP-Glc, UDP-Gal and UDP-Xyl reactions, respectively), 1.0 mM of saturating phloretin, 10 mM 2-mercaptoethanol, and different concentrations of UDP-sugars.

References

Li, T., Borg, A. J. E., Krammer, L., Breinbauer, R., & Nidetzky, B. (2023). Reaction intensification for biocatalytic production of polyphenolic natural product di-*C*-β-glucosides. *Biotechnology and Bioengineering*, *120,* 1506-1520. https://doi.org/10.1002/bit.28354

Savino, S., Borg, A. J. E., Dennig, A., Pfeiffer, M., De Giorgi, F., Weber, H., Dubey, K. D., Rovira, C., Mattevi, A., & Nidetzky, B. (2019). Deciphering the enzymatic mechanism of sugar ring contraction in UDP-apiose biosynthesis. *Nature Catalysis*, *2,* 1115-1123. https://doi.org/10.1038/s41929-019-0382-8

Schmölzer, K., Lemmerer, M., & Nidetzky, B. (2018). Glycosyltransferase cascades made fit for chemical production: Integrated biocatalytic process for the natural polyphenol C-glucoside nothofagin. *Biotechnology and Bioengineering, 115*, 545-556. https://doi.org/10.1002/bit.26491

Zhang, M., Li, F. D., Li, K., Wang, Z. L., Wang, Y. X., He, J. Bin, Su, H. F., Zhang, Z. Y., Chi, C. B., Shi, X. M., Yun, C. H., Zhang, Z. Y., Liu, Z. M., Zhang, L. R., Yang, D. H., Ma, M., Qiao, X., & Ye, M. (2020). Functional characterization and structural basis of an efficient di-*C*-glycosyltransferase from *Glycyrrhiza glabra*. *Journal of the American Chemical Society*, *142*, 3506-3512. https://doi.org/10.1021/jacs.9b12211
